# Structural Phase Transformations Induced by Guest Molecules in a Nickel-Based 2D Square Lattice Coordination Network

**DOI:** 10.1021/acs.chemmater.2c03662

**Published:** 2023-01-10

**Authors:** Xia Li, Debobroto Sensharma, Varvara I. Nikolayenko, Shaza Darwish, Andrey A. Bezrukov, Naveen Kumar, Wansheng Liu, Xiang-Jing Kong, Zhenjie Zhang, Michael J. Zaworotko

**Affiliations:** † Department of Chemical Science, Bernal Institute, University of Limerick, Limerick V94 T9PX, Republic of Ireland; ‡ College of Chemistry, Nankai University, Tianjin 300071, People’s Republic of China

## Abstract

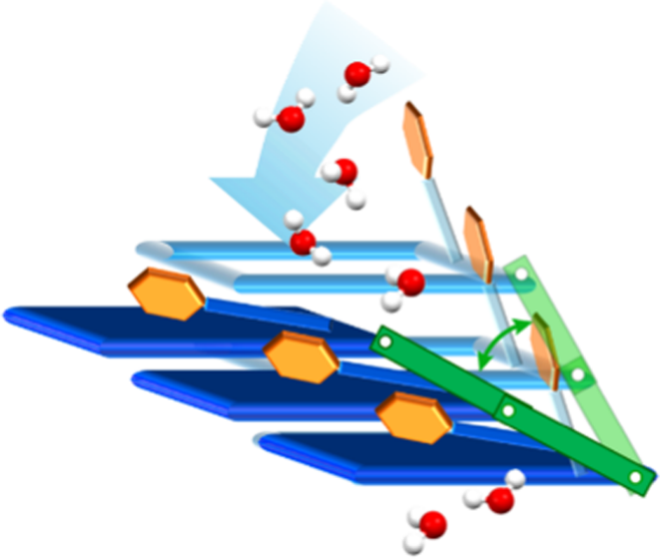

Herein, we report the crystal structure and guest binding properties of a new two-dimensional (2D) square lattice (**sql**) topology coordination network, **sql-(azpy)(pdia)-Ni**, which is comprised of two linker ligands with diazene (azo) moieties, (*E*)-1,2-di(pyridin-4-yl)diazene(azpy) and (*E*)-5-(phenyldiazenyl)isophthallate(pdia). **sql-(azpy)(pdia)-Ni** underwent guest-induced switching between a closed (nonporous) **β** phase and several open (porous) **α** phases, but unlike the clay-like layer expansion to distinct phases previously reported in switching **sql** networks, a continuum of phases was formed. In effect, **sql-(azpy)(pdia)-Ni** exhibited elastic-like properties induced by adaptive guest binding. Single-crystal X-ray diffraction (SCXRD) studies of the **α** phases revealed that the structural transformations were enabled by the pendant phenyldiazenyl moiety on the pdia^2–^ ligand. This moiety functioned as a type of hinge to enable parallel slippage of layers and interlayer expansion for the following guests: *N*,*N*-dimethylformamide, water, dichloromethane, *para*-xylene, and ethylbenzene. The slippage angle (interplanar distances) ranged from 54.133° (4.442 Å) in the **β** phase to 69.497° (5.492 Å) in the ethylbenzene-included phase. Insight into the accompanying phase transformations was also gained from variable temperature powder XRD studies. Dynamic water vapor sorption studies revealed a stepped isotherm with little hysteresis that was reversible for at least 100 cycles. The isotherm step occurred at ca. 50% relative humidity (RH), the optimal RH value for humidity control.

## Introduction

Since “third generation coordination polymers”^1^ or “soft porous crystals”^2^ were introduced in the late 1990s and early 2000s, flexible metal–organic materials (FMOMs), i.e., materials that adjust their structures when exposed to external stimuli, have been explored with emphasis upon their potential utility for gas, vapor, and liquid storage applications.^3−7^ Whereas rigid porous coordination networks (PCNs) typically display type I (Langmuir) sorption isotherms and some microporous PCNs can exhibit exceptional selectivity in the context of separation of industrially relevant gas and vapor mixtures, such as C1 gases;^8^ C2 gases;^9,10^ C3 gases;^11,12^ C6 aromatics;^13^ and C8 aromatics,^14^ FMOMs can undergo structural transformation(s) in response to guest molecules and sometimes exhibit sharp-stepped isotherms that are usually accompanied by a phase transformation from a closed (nonporous) to an open (porous) phase (switching).^15−19^ The ability of FMOMs to adjust their pore geometry as a consequence of structural transformations^20−22^ can enable enhanced working capacity and thermodynamic management, which is relevant for gas storage applications.^23^

An archetypal class of PCNs is the family of coordination networks with square lattice (**sql**) topology, the prototypal variant of which was reported in 1970.^24^ In the 1990s, **sql** networks involving 4,4′-bipyridine, bpy, were introduced with both interpenetrated^25^ and noninterpenetrated^26^ variants. These **sql** networks are highly amenable to crystal engineering^27^ thanks to their modularity in terms of the metal, linker ligands, and for octahedral metal centers, the terminal ligand.^28−30^ Our analysis of the TOPOS topological types observed database^31^ and Cambridge Structural Database^32^ (TOPOS TTO ∩ CSD databases, see Supporting Information for details) revealed that there are >9000 **sql** network examples (Figure S1). Out of these, >2500 **sql** networks have been reported involving N-donor linker ligands, including both single-linker and mixed-linker **sql** networks.^33^

An interesting feature of **sql** networks is that when they form layered structures, they can exhibit switching transformations between closed and open phases. The structure of the prototypal switching **sql** network, [Cu(bpy)_2_(BF_4_)_2_], **ELM-11**, was reported in 2001.^34^**ELM-11**^35,36^ and its variant, **ELM-12**,^30^ [Cu(bpy)_2_(OTf)_2_], OTf = triflate, have been widely studied along with other bpy linked **sql** analogues.^37^ More recently, [Co(bipy)_2_(NCS)_2_], **sql-1-Co-NCS**, set a new performance benchmark for C8 hydrocarbon separations in terms of both selectivity and uptake.^17^ The mechanism of switching in such networks can be attributed to clay-like expansion/shrinkage between adjacent layers of **sql** planes and typically results in distinct phases supported by interlayer interactions in addition to layer–guest interactions.^28,38^ Typically, there is little deformation or flexibility associated with the **sql** network itself.

**Figure 1:**
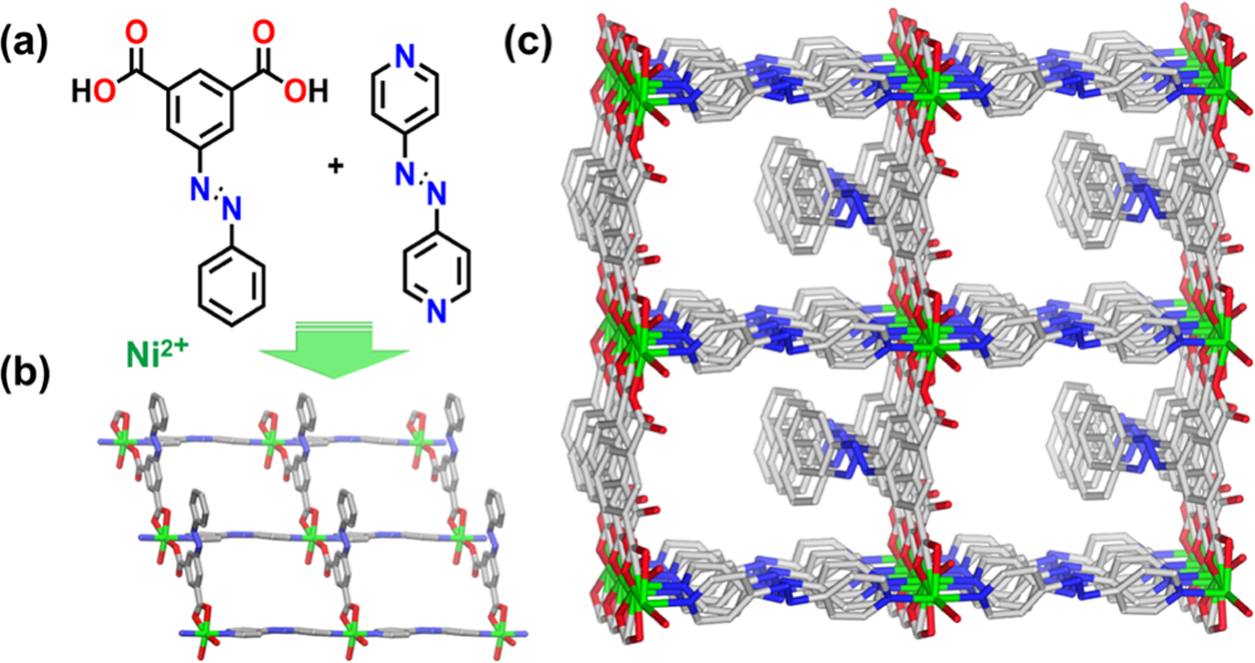
(a) Ligands (*E*)-5-(phenyldiazenyl) isophthalic acid (H_2_pdia, left) and (*E*)-1,2-di(pyridin-4-yl)diazene (azpy, right). (b) **sql** layer in **sql-(azpy)(pdia)-Ni**. (c) One-dimensional (1D) channels lie along the *c*-axis in the as-synthesized form (disordered guests are omitted for the sake of clarity), **sql-(azpy)(pdia)-Ni-α**_**DMF**_.

Ligands with diazene (azo) moieties are known to enable deformation and so offer the potential for additional flexibility.^39−41^ Thus far, only four structures have been reported based upon H_2_pdia = (*E*)-5-(phenyldiazenyl)isophthalic acid, H_2_pdia (Figure 1a), but these studies did not focus on sorption, and none were reported to be flexible.^42,43^ In TOPOS TTO ∩ CSD databases, 55 **sql** networks based upon (*E*)-1,2-di(pyridin-4-yl)diazene, azpy (Figure 1a) linkers have been reported (Figure S2 and Table S1), 26 of which are single-linker **sql** nets (Type I-a),^33^ whereas 19 examples are mixed-linker **sql** nets with dicarboxylate anions as the second linker (Type II-ab).^33^ Although **sql** nets based on two distinct azo-bearing linkers^44^ and flexible **sql** nets featuring azo-functionality^45^ have been studied, to the best of our knowledge, the flexibility of the diazene moiety has not been exploited to induce switching behavior.

In this study, we report what is to our knowledge, the first example of a switching **sql** network sustained by mixed-linker ligands that both contain azo moieties, **sql-(azpy)(pdia)-Ni** ([Ni(pdia)(azpy)(H_2_O)], Figure 1a), its unusual guest-induced switching behavior, and characterization of that switching behavior with emphasis upon the effect of the pendant azo moiety of the pdia^2–^ linker ligand upon the nature of the observed structural changes.

## Experimental Section

### **sql-(azpy)(pdia)-Ni-α_DMF_**, {[Ni(azpy)(pdia)(H_2_O)]·DMF}

#### Synthesis

A mixture of Ni(NO_3_)_2_·6H_2_O (0.15 mmol, 43.5 mg), (*E*)-1,2-di(pyridin-4-yl)diazene (azpy) (0.15 mmol, 27.6 mg), (*E*)-5-(phenyldiazenyl)isophthalic acid (H_2_pdia) (0.15 mmol, 40.5 mg), *N*,*N*-dimethylformamide (DMF) (5.0 mL), and water (H_2_O) (5.0 mL) was added to a 20 mL glass vial. The vial was capped tightly and placed in an oven at 105 °C for 24 h, which was then cooled to room temperature. After rinsing several times with fresh DMF, brown single crystals were obtained. Yield: 78%. IR: ν_max_ (cm^–1^) = 3392, 3292, 2934, 2868, 1650, 1596, 1524, 1388, 1219, 1096, 776, 718.

### **sql-(azpy)(pdia)-Ni-β**, [Ni(azpy)(pdia)(H_2_O)]

#### Synthesis

The as-synthesized open framework (**sql-(azpy)(pdia)-Ni-α**_**DMF**_) was exchanged with fresh methanol (MeOH) using a Soxhlet extractor for 2 days and then heated to 100 °C under vacuum for 10 h to yield **sql-(azpy)(pdia)-Ni-β**. IR: ν_max_ (cm^–1^) = 3363, 1593, 1557, 1531, 1353, 1222, 1044, 1019, 918, 832, 761, 715.

### **sql-(azpy)(pdia)-Ni-α_H_2_O_**, {[Ni(azpy)(pdia)(H_2_O)]·3H_2_O}

#### Synthesis

The activated closed framework (**sql-(azpy)(pdia)-Ni-β**) was soaked in water for 1 day to yield **sql-(azpy)(pdia)-Ni-α**_**H_2_O**_. IR: ν_max_ (cm^–1^) = 3326, 1596, 1553, 1530, 1356, 1222, 1144, 1099, 1050, 1015, 918, 778, 721, 684.

### **sql-(azpy)(pdia)-Ni-α_DCM_**, {[Ni(azpy)(pdia)(H_2_O)]·0.5DCM}

#### Synthesis

The activated closed framework (**sql-(azpy)(pdia)-Ni-β**) was soaked in dichloromethane (DCM) for 1 day to yield **sql-(azpy)(pdia)-Ni-α**_**DCM**_.

### **sql-(azpy)(pdia)-Ni-α_PX_**, {[Ni(azpy)(pdia)(H_2_O)]·0.5PX}

#### Synthesis

The activated closed framework (**sql-(azpy)(pdia)-Ni-β**) was soaked in *para*-xylene (PX) for 1 day to yield **sql-(azpy)(pdia)-Ni-α**_**PX**_.

### **sql-(azpy)(pdia)-Ni-α_EB_**, {[Ni(azpy)(pdia)(H_2_O)]·0.58407EB}

#### Synthesis

The activated closed framework (**sql-(azpy)(pdia)-Ni-β**) was soaked in ethylbenzene (EB) for 1 day to yield **sql-(azpy)(pdia)-Ni-α**_**EB**_.

## Dynamic Vapor Sorption (DVS) Experiments

Dynamic water vapor sorption studies were performed on ca. 10 mg samples using a Surface Measurement Systems Adventure Dynamic Vapor Sorption (DVS) system, which gravimetrically measures the uptake and loss of vapor using air as a carrier gas. Pure water was used as the adsorbate for these measurements, and temperature was maintained at 298 K by enclosing the system in a temperature-controlled incubator. The mass of the sample was determined by comparison to an empty reference pan and recorded by a high-resolution microbalance with a precision of 0.01 μg. Sorption isotherms were measured from 0 to 95% relative humidity (RH) stepwise with a convergence equilibrium criterion d*m*/d*t* = 0.01%/min. The minimum and maximum equilibration times for each step were 10 and 360 min, respectively.

## Results and Discussion

**Figure 2:**
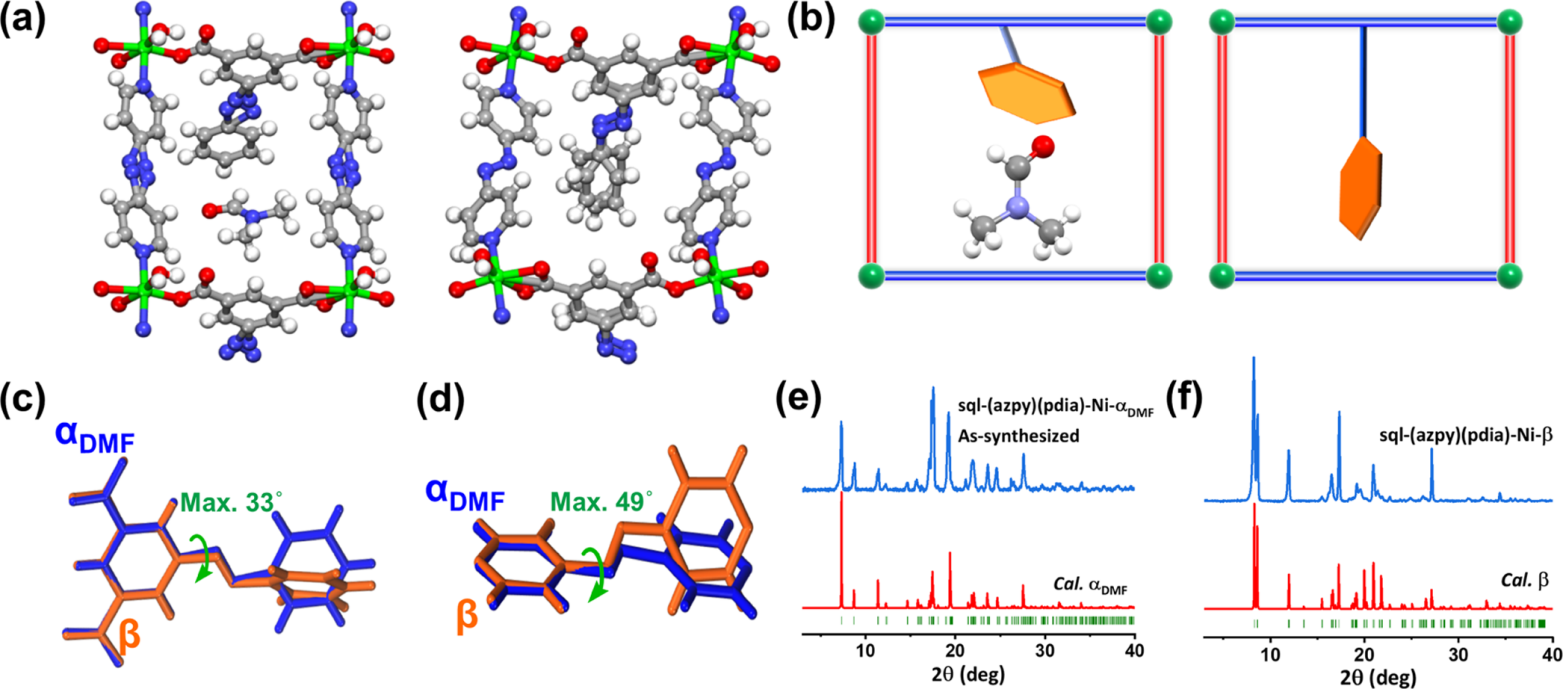
(a) **sql** nets in the **α_DMF_** and **β** phases of **sql-(azpy)(pdia)-Ni**. (b) Schematic diagram of **α**_**DMF**_ and **β**. Superposed representations of deformations in (c) pdia^2–^ and (d) azpy in **α**_**DMF**_ (blue) and **β** (orange). Comparison of experimental PXRD patterns of (e) **α**_**DMF**_ and (f) **β** and PXRD patterns calculated from the SCXRD determined structures.

Single crystals of **sql-(azpy)(pdia)-Ni** were obtained by solvothermal reaction of H_2_pdia and azpy in DMF and water at 105 °C, yielding **sql-(azpy)(pdia)-Ni-α**_**DMF**_. Single-crystal X-ray diffraction (SCXRD) was used to determine the crystal structure of **sql-(azpy)(pdia)-Ni-α**_**DMF**_, which had crystallized in the monoclinic space group *Pc* with *a* = 13.2072(4)4 Å, *b* = 10.1554(3) Å, *c* = 11.2001(4)4 Å, α = γ = 90°, β = 113.920(10)°, and *V* = 1373.19(8) Å^3^ (Table S2). The octahedral mononuclear molecular building block (MBB)^46^ is comprised of a Ni^2+^ cation coordinated to two N-donor atoms (N1 and N4) from two azpy ligands, three carboxylate O-donor atoms (O1, O3, and O4) from two pdia^2–^ ligands, and one O-donor atom (O5) from an aqua ligand. The formula is [Ni(pdia)(azpy)(H_2_O)]·DMF (Figure S3). Hydrogen bonds were observed between each coordinated aqua molecule and O-atoms of coordinated pdia^2–^ ligands, both within individual **sql** layers (O···O = 2.614(5) Å) and between neighboring **sql** layers (O···O = 2.783(6) Å, Table S3). A 3D network with primitive cubic, **pcu**, topology structure results. The perpendicular distance between adjacent **sql** planes formed by Ni cations is 5.120(6) Å, and the dihedral angle between the **sql** plane and the plane of the parallelogram formed from two pairs of Ni^2+^ cations from adjacent **sql** layers is 66.075(13)° (Table S3). **sql-(azpy)(pdia)-Ni-α**_**DMF**_ has an effective pore size of ca. 6 × 7 Å^2^ along the *c*-axis, and the calculated guest-accessible void volume is 19.5% (Figures 1c, S4, and S5). The bulk experimental powder X-ray diffraction (PXRD) pattern of **α**_**DMF**_ is consistent with that calculated from SCXRD data (Figure 2e).

Prior to conducting gas sorption experiments, **sql-(azpy)(pdia)-Ni-α**_**DMF**_ was soaked in methanol, exchanged twice daily for 2 days, and then activated at 100 °C under vacuum. The activated phase had transformed to a nonporous phase, **sql-(azpy)(pdia)-Ni-β**, as determined by SCXRD. **sql-(azpy)(pdia)-Ni-β** is a contorted version of **sql-(azpy)(pdia)-Ni-α**_**DMF**_ with the same connectivity and space group but different unit-cell parameters and a 14.6% reduction in unit-cell volume when compared to **α**_**DMF**_ (Table S2). The **α**_**DMF**_ to **β** transformation was accompanied by a folding motion of the channels along the *c*-axis, resulting in reduction of guest-accessible volume from 19.5% (**α**_**DMF**_) to 0% (**β**) as calculated by Mercury software (Figure S4). The transformation from **α**_**DMF**_ and **β** can be attributed to a deformation in the pdia^2–^ and azpy ligands whereby “hinge-like” rotation occurred along the azo bond. In effect, the azo bonds in pdia^2–^ and azpy act as axles (Figure 2a,b). Close contacts in **β** were found between two interlayer pdia^2–^ linkers, which drives the change of **α**_**DMF**_ to a denser phase on guest removal (Figure S6, *d*_C21A···N6_ = 3.42 Å, *d*_C20A···N5_ = 3.43 Å). The dihedral angles of the isophthalate ring and phenyl ring of pdia^2–^ ligand in **α**_**DMF**_ and **β** changed substantially from 8.161 to 48.930° (47.373°) (Table S4). The azo bond, which connects the two pyridine rings in azpy ligand, also changes orientation during the transformation from **α**_**DMF**_ to **β**, the dihedral angles between these two pyridine rings being 4.756 and 69.133°, for **α**_**DMF**_ and **β**, respectively (Table S4).

**Figure 3:**
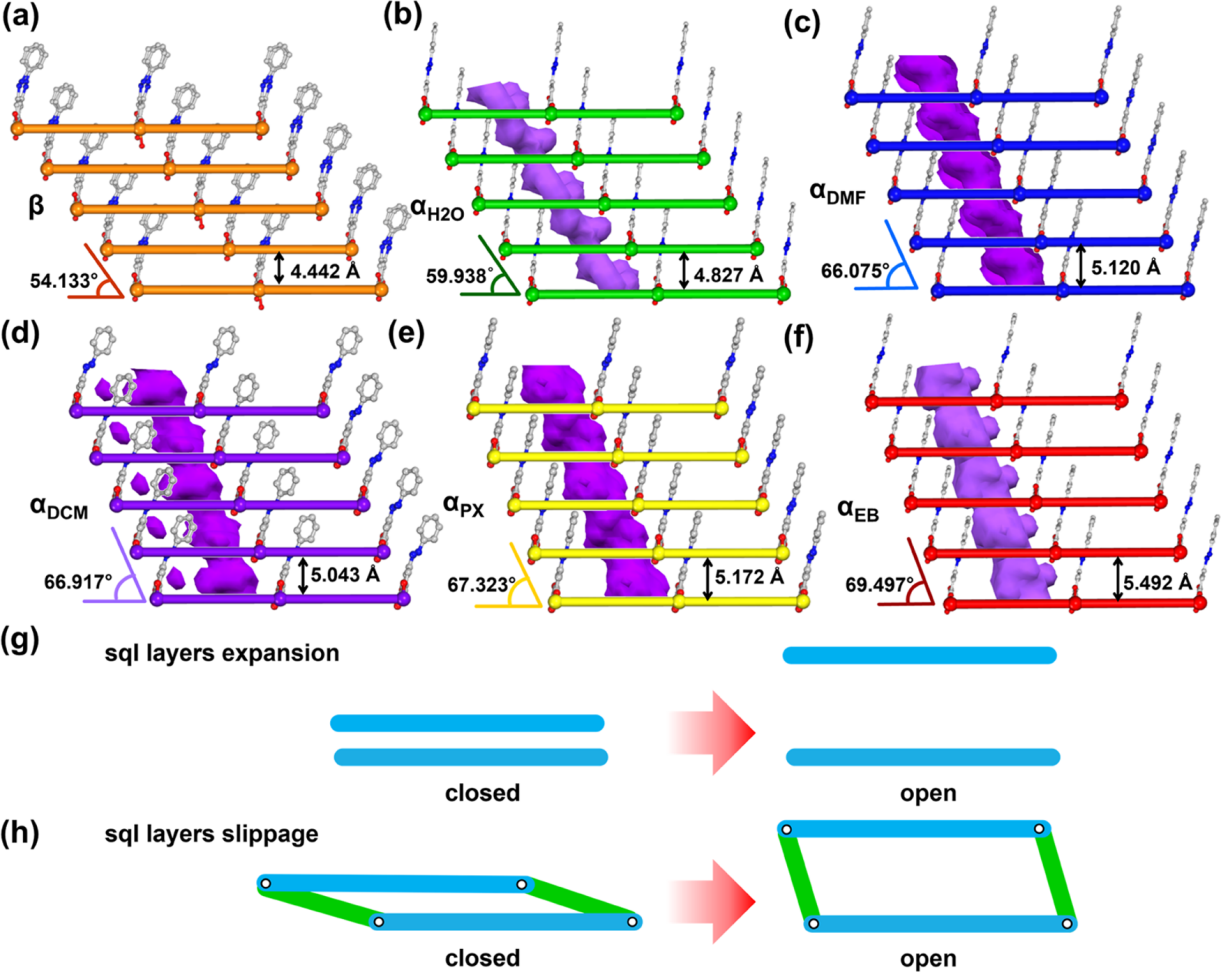
Schematic representation of the slippage and expansion of layers (rods = azpy linkers, balls = Ni^2+^ cations) and voids (purple channels) in the phases of **sql-(azpy)(pdia)-Ni** studied herein: (a) **sql-(azpy)(pdia)-Ni-β** (nonporous); (b) **sql-(azpy)(pdia)-Ni-α**_**H_2_O**_; (c) **sql-(azpy)(pdia)-Ni-α**_**DMF**_; (d) **sql-(azpy)(pdia)-Ni-α**_**DCM**_; (e) **sql-(azpy)(pdia)-Ni-α**_**PX**_; and (f) **sql-(azpy)(pdia)-Ni-α**_**EB**_. The mechanism of (g) **sql** layer expansion in previously reported **sql** networks and (h) **sql** layer slippage/expansion seen herein (**sql** layers = blue, hydrogen bonds = green).

When the isophthalate rings of pdia^2–^ in **α**_**DMF**_ and **β** are superposed and compared, the maximum torsional angle about the azo bond is ca. 33° (Figure 2c). Upon superposing the corresponding pyridine rings of azpy in **α**_**DMF**_ and **β**, the torsional angle about the azo bond was found to be ca. 49° (Figure 2d). These two rotations work synergistically to shrink the voids in **β** and are enabled by the pendant pdia^2–^ ligand (Figures 2a,b). The experimental PXRD pattern of **β** is consistent with that calculated from SCXRD data (Figure 2f). The interlayer H-bond between **α**_**DMF**_ and **β** hardly changed (2.783(6)–2.832(7) Å), but the distance between adjacent **sql** planes in **α**_**DMF**_ and **β** decreased from 5.120(6) to 4.442(10) Å (Figure 3c,a). The dihedral angle between the **sql** plane and the parallelogram formed by pairs of Ni^2+^ cations from adjacent layers reduced from 66.075(13) to 54.133(21)° (Table S3). In effect, slippage between **sql** layers had occurred during the transformation from **α**_**DMF**_ to **β**.

To further explore the mechanism of flexibility in **sql-(azpy)(pdia)-Ni**, crystals of **sql-(azpy)(pdia)-Ni-β** were soaked in the following solvents: H_2_O, DCM, PX, and EB. SCXRD experiments revealed that four additional open phases were obtained, namely, **sql-(azpy)(pdia)-Ni-α**_**H_2_O**_, **sql-(azpy)(pdia)-Ni-α**_**DCM**_, **sql-(azpy)(pdia)-Ni-α**_**PX**_, and **sql-(azpy)(pdia)-Ni-α**_**EB**_. SCXRD data revealed that folding/unfolding of the pores and slippage between layers had occurred in such a manner that **sql-(azpy)(pdia)-Ni**, in effect, adapts its structure for each guest. Cell volumes were as follows: **β** = 1199.07(12) Å^3^; **α**_**H_2_O**_ = 1296.43(5) Å^3^; **α**_**DCM**_ = 1350.30(7) Å^3^; **α**_**DMF**_ = 1373.19(8) Å^3^; **α**_**PX**_ = 1389.00(9) Å^3^; and **α**_**EB**_ = 1469.2(3) Å^3^ (Table S2). These cell volumes correspond to the relative molecular volume of each guest: H_2_O (2 × 18.4 Å^3^); DCM (1 × 58 Å^3^); DMF (1 × 72.3 Å^3^); PX (1 × 110.2 Å^3^); and EB (1 × 110.2 Å^3^), as calculated by XSeed,^47^ except for H_2_O, which was calculated from the CSD.^48,49^ Although PX and EB exhibit the same molecular volume, the shape of PX enabled a better fit than EB, which in turn required a larger pore volume. Short contact distances between guest molecules and pore walls were observed for **α**_**DMF**_ (*d*_(O···H)_ = 2.516 Å), **α**_**H_2_O**_ (*d*_(O···O)_ = 2.969 Å), **α**_**EB**_ (*d*_(C–H···π)_ = 3.005 Å), **α**_**DCM**_ (*d*_(O···H)_ = 2.294 Å), and **α**_**PX**_ (*d*_(O···H)_ = 2.927 Å) (Table S3). Disorder of ligands was observed in **α**_**DMF**_, **β**, **α**_**H_2_O**_, and **α**_**EB**_ related to host–guest interactions and framework flexibility. Overall, the analysis of the crystal structures of the six phases indicates that adaptive binding (Figures 3a–f and S5) and slippage motion are enabled by the pendant phenyldiazenyl moiety of the pdia^2–^ ligand.

The continuous nature of the slippage/expansion in **sql-(azpy)(pdia)-Ni** can be quantified by the **sql** layer separation and dihedral angle formed by the **sql** plane and the plane formed by pairs of Ni^2+^ cations from adjacent **sql** layers (Table S3). At one extreme, **α**_**EB**_ possesses the biggest void volume (21.8%), longest interlayer distance (5.492(15) Å), and most obtuse dihedral angle (69.497(20)°) (Figure 3f). At the other extreme, **β** is nonporous (0%) and has the shortest interlayer distance (4.442(10) Å) and the most acute dihedral angle (54.133(26)°) (Figure 3a). The interlayer hydrogen bonds in all six phases lie within a narrow range, 2.74(8)–2.83(7) Å (Table S3), whereas the other structural parameters are intermediate between the extremes. The motion between **sql** layers can be described as being analogous to parallel motion linkage, a concept from engineering and architecture (Figure 3h).^50^

Layer expansion in previously reported **sql** networks like **ELM-11**,^36^**ELM-12**,^51^ and **sql-1-Co-NCS**(17) resulted from phase transformations between two or more discrete phases (Figure 3g). In **ELM-11**, the expansion of **sql** layers was induced by carbon dioxide (CO_2_),^36^*n*-butane,^52^ and acetylene (C_2_H_2_).^53^ With increasing pressure of CO_2_, the phase change from closed to open phases resulted in distances between **sql** layers of 4.427, 5.676, 5.685, and 6.960 Å. In **ELM-12**, vacuum heating prompted interlayer **sql** distances to decrease from 7.2 to 6.7 Å and then to 5.9 Å, corresponding to three distinct phases. C8 aromatics were reported to induce **sql-1-Co-NCS** to exhibit four distinct phases with interlayer **sql** distances of 4.46 (closed) to 9.15 (PX), 9.21 (*meta*-xylene, MX), 9.26 (*ortho*-xylene, OX), and 6.25 (EB) Å. In contrast, anchored by an interlayer H-bond, layer expansion was not as substantial in **sql-(azpy)(pdia)-Ni**. Rather, slippage of **sql** layers was induced by a series of guests to increase molecular volume (Figure 3h) accompanied by phase transformations driven by guest size.

The bulk phase purities of **β** and the five solvates reported herein were confirmed by matching of experimental and calculated PXRD patterns (Figures 2e,f and S7). Further, PXRD experiments conducted after immersing **sql-(azpy)(pdia)-Ni-β** in aqueous solutions with a range of pH values revealed that **sql-(azpy)(pdia)-Ni** retained its crystallinity after exposure to pH values ranging from 2 to 11 (Figure S8). Crystallinity was also retained under accelerated humidity stability testing conditions over 48 h (45 °C, 95% relative humidity, RH, Figure S9). The thermal stability of **sql-(azpy)(pdia)-Ni** was evaluated by thermogravimetric analysis (TGA) and variable temperature PXRD (VT-PXRD). TGA results revealed that **sql-(azpy)(pdia)-Ni-α**_**DMF**_ exhibited a mass loss of 11.4% at 115 °C, corresponding to one DMF molecule per formula unit (calculated 12.1%). TGA conducted on **sql-(azpy)(pdia)-Ni-β** showed no mass loss below thermal decomposition at 513 K (Figure S10). Attempts to analyze **α**_**H_2_O**_ by TGA showed identical results to **β**, which indicated desorption of water at ambient conditions (indoor humidity < 40%, Figure S10). VT-PXRD conducted on **sql-(azpy)(pdia)-Ni-α**_**H_2_O**_ revealed that a transformation to **β** occurred at 298 K at the onset of nitrogen (N_2_) flow without application of heat, following which **β** was found to be stable up to 473 K (Figure S11).

Encouraged by the stability and responsive solution-phase uptake behavior of **sql-(azpy)(pdia)-Ni**, we studied its gas sorption properties. **sql-(azpy)(pdia)-Ni-β** revealed negligible uptake for N_2_ at 77 K, whereas a stepped isotherm was observed for CO_2_ at 195 K, with the inflection occurring at very low pressure (*P*/*P*_0_ = 0.01, uptake 76 cm^3^/g, Figure S12), confirming that **sql-(azpy)(pdia)-Ni** acts as a stimulus-responsive sorbent. In addition, the CO_2_ sorption at 273 K exhibited the onset of a step at 681 mmHg, which is consistent with a structural transformation (Figure S12). This step was not observed below 1 bar at 298 K. The single point pore volume calculated at saturation (*P*/*P*_0_ = 0.9) from the 195 K CO_2_ isotherm is 0.118 cm^3^ g^–1^, which agrees well with the crystallographically determined accessible void volume in **sql-(azpy)(pdia)-Ni-α**_**H_2_O**_ (0.124 cm^3^/g), indicating that the CO_2_-included phase resembles **α**_**H_2_O**_.

**Figure 4:**
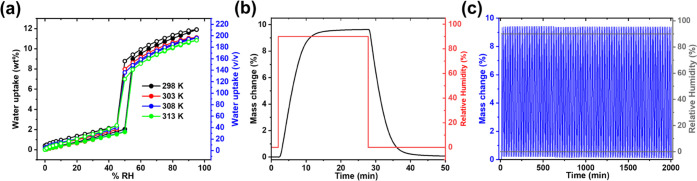
(a) Dynamic water vapor sorption isotherms of **sql-(azpy)(pdia)-Ni** at different temperatures (298, 303, 308, and 313 K). (b) Dynamic water vapor adsorption–desorption kinetic curves of **sql-(azpy)(pdia)-Ni** on 10.6 mg of sample at 298 K. (c) 100 cycles of dynamic adsorption–desorption water sorption of **sql-(azpy)(pdia)-Ni** between 0 and 90% RH on 10.4 mg of sample at 298 K.

**Figure 5:**
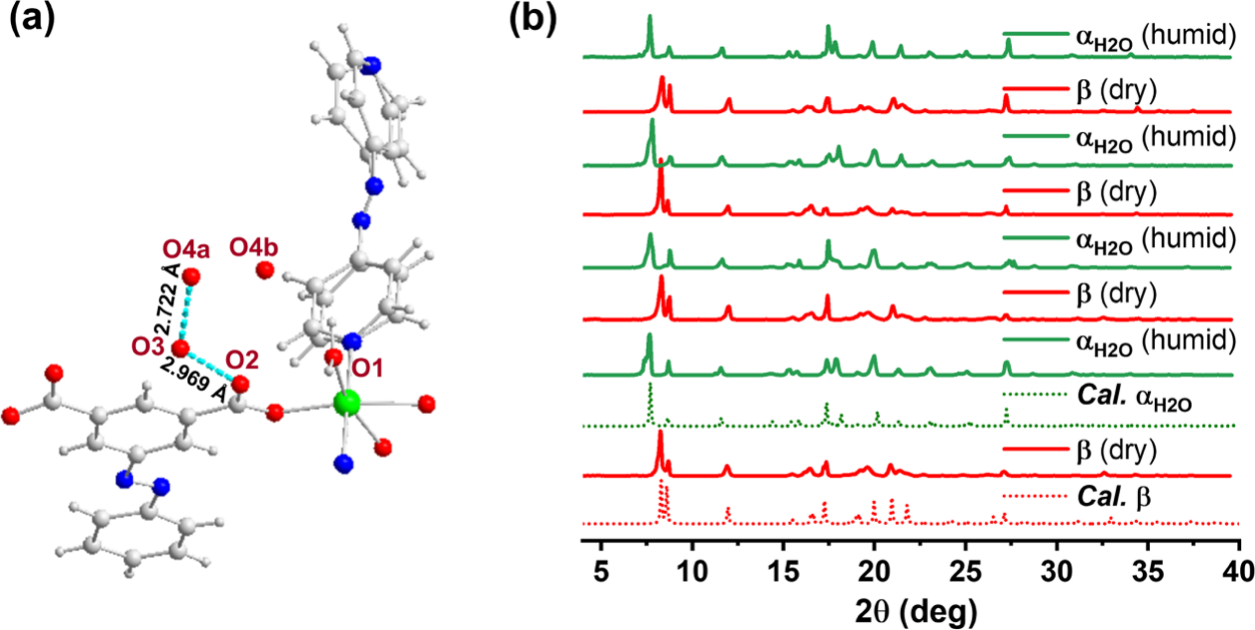
(a) Close contacts (cyan lines) between water molecules and ligand H_2_pdia in **sql-(azpy)(pdia)-Ni-α**_**H_2_O**_. (b) Overlaid PXRD patterns showing the reversible change between **sql-(azpy)(pdia)-Ni-α**_**H_2_O**_ and **sql-(azpy)(pdia)-Ni**-**β** under humidity swing conditions.

That water can serve as a guest in **sql-(azpy)(pdia)-Ni** is of topical interest since water sorbents are being studied for their potential utility in water harvesting and dehumidification.^54,55^ To evaluate the sorption characteristics of **sql-(azpy)(pdia)-Ni**, dynamic water vapor sorption experiments were conducted on **sql-(azpy)(pdia)-Ni-β**. A stepped isotherm with an abrupt uptake was observed at 298 K. The step occurred between 50 and 55% RH with an uptake of 2 wt % (36 cm^3^/cm^3^, cc/cc) at 50% RH and an uptake of 9 wt % (162 cc/cc) at 55% RH. The uptake difference of 7 wt % (126 cc/cc) is consistent with two water molecules per formula unit (6.8 wt %, Figure 4a). This value is in agreement with the observation of two water molecules (O3 is fully occupied, while O4 is disordered over two positions; O4a/O4b is 0.43:0.57) in the crystal structure of **sql-(azpy)(pdia)-Ni**-**α**_**H_2_O**_, which exhibits both water–water and water–framework short contacts (Figure 5a). Including the contribution from surface uptake, the saturation water uptake at 95% RH was found to be 12 wt % (216 cc/cc, Figure 4a).

In contrast to the pore filling (Type V) mechanism, which is often observed in rigid sorbent frameworks, such a stepped isotherm, along with the aforementioned structural changes, indicates that a nonporous-to-porous switching event occurs from **β** to **α**_**H_2_O**_ during the adsorption of water. The desorption isotherm shows little hysteresis with steep water desorption at ca. 45% RH (Figure 4a). This kind of stepped sorption isotherm wherein the adsorption and desorption branches are centered between 40 and 60% RH range, but with distinct gate-opening and gate-closing partial pressures, is ideal for an autonomous moisture-controlled swing process.^55−59^ Moreover, upon increasing the sorption temperature from 298 to 303, 308, and 313 K, the relative gate-opening pressure hardly changed, and there was little loss of working capacity (313 K uptake of 11 wt % or 198 cc/cc), suggesting a wide operating temperature range for indoor humidity control.

The sorption kinetics of **sql-(azpy)(pdia)-Ni-β** were studied by subjecting a sample to humidity swing conditions, wherein RH was varied between 0 to 90%. As shown in Figure 4b, **sql-(azpy)(pdia)-Ni** can not only rapidly capture water vapor from the gas phase (as much as 9.4 wt % in 10 min or 169.2 cc/cc at 90% RH and 298 K) but also can be fully regenerated in less than 10 min once the RH is decreased to 0%. To evaluate the reusability of **sql-(azpy)(pdia)-Ni** for water sorption, humidity swing conditions of 298 K, 0–90% RH were applied to the sample for 100 cycles of 20 min each (10 min adsorption, 10 min desorption). No loss of working capacity was observed over the course of the experiment despite undergoing structural transformations during each cycle (Figure 4c). The recovered **β** phase retained crystallinity after the 100th cycle, as confirmed by PXRD analysis (Figure S13). To approximate real-world dehumidification conditions, sorption kinetics from 40 to 60% were also studied. The uptake was found to reach 9 wt % (162 cc/cc) within 60 min at 60% RH, and desorption to a loading of 1 wt % (18 cc/cc) occurred within 25 min at 40% RH. Working capacity was not significantly reduced over 12 cycles under these conditions (Figure S14).

The mechanism of water sorption by the switching of **sql-(azpy)(pdia)-Ni** was further studied by monitoring its structure by PXRD during repeated exposure to dry and humid conditions. First, **sql-(azpy)(pdia)-Ni** was exposed to dry N_2_ flow at room temperature (10 min), exhibiting a PXRD pattern matching with the **β** phase. Then, instead of N_2_ flow, a PXRD pattern of **sql-(azpy)(pdia)-Ni** was measured in a humid atmosphere at the same temperature. **α**_**H_2_O**_ was obtained, as expected. PXRD patterns corresponded to the **β** and **α**_**H_2_O**_ phases without loss of crystallinity over 4 cycles of the experiment (Figure 5b). In addition, single crystals randomly taken from a bulk sample of **sql-(azpy)(pdia)-Ni-β** and exposed to successive hydration and evacuation showed corresponding changes from **β** to **α**_**H_2_O**_ and back to **β** did not exhibit signs of fragmentation despite somewhat diminished crystal quality (Table S2). Time-lapse spectra Fourier Transform Infrared (FTIR) studies support the observed fast kinetics of water loading and unloading, showing a progressive reduction of the strong O–H water stretching peak at 3326 cm^–1^ until its disappearance within 10 min under ambient conditions (20 °C, 40% RH, Figure S15), leaving only peaks due to coordinated water in **β** (3356 cm^–1^). These data reveal that regeneration of **sql-(azpy)(pdia)-Ni** can be easily realized by simply adjusting RH. Favorable intrinsic heat management arising from the endothermic structural transformation from **β** to **α**_**H_2_O**_ is anticipated. To date, over 370 desiccant MOMs have been investigated, but fewer than 10% exhibit water-induced flexibility (Table S5), and, to our knowledge, no two-dimensional (2D) FMOMs have previously been studied for humidity control. **sql-(azpy)(pdia)-Ni** is therefore a potential candidate for indoor humidity control.

## Conclusions

In conclusion, a new switching 2D MOF **sql-(azpy)(pdia)-Ni** was synthesized using the azpy and pendant-bearing pdia^2–^ ligands. Adaptive binding of guest molecules resulted in six distinct phases of **sql-(azpy)(pdia)-Ni** corresponding to phases loaded with DMF, H_2_O, DCM, PX, EB, and a nonporous phase, as elucidated by SCXRD and PXRD. The continuum of phases exhibited by **sql-(azpy)(pdia)-Ni** was facilitated by the pendant phenyldiazenyl moiety on the pdia^2–^ ligand and interlayer hydrogen bonds between adjacent **sql** layers. This elastic-like motion is key to regulating access to the pores in each phase and was also observed during gas and H_2_O sorption. In particular, the stepped water sorption isotherm with inflections in the range 40–60% RH makes **sql-(azpy)(pdia)-Ni** a potential candidate for indoor humidity control applications. Studies of stability and kinetics on **sql-(azpy)(pdia)-Ni** show excellent recyclability and retention of crystallinity. We attribute the elastic-like properties of **sql-(azpy)(pdia)-Ni** to the characteristic of rotation in diazo moieties and the pendant nature of one of the linker ligands, which enables a hinge-like mechanism of structural transformation. The amenability of **sql** nets to crystal engineering approaches will likely afford more members of this family of switching sorbent materials.

## Supporting Information Available

Materials and methods; supporting figures; supporting tables; and supporting references (PDF)
X-ray data for **sql-(azpy)(pdia)-Ni-α_DMF_** (CIF)
X-ray data for **sql-(azpy)(pdia)-Ni-β** (CIF)
X-ray data for **sql-(azpy)(pdia)-Ni-β′** (CIF)
X-ray data for **sql-(azpy)(pdia)-Ni-α_DCM_** (CIF)
X-ray data for **sql-(azpy)(pdia)-Ni-α_H_2_O_** (CIF)
X-ray data for **sql-(azpy)(pdia)-Ni-α_PX_** (CIF)
X-ray data for **sql-(azpy)(pdia)-Ni-α_EB_** (CIF)

## Supplementary Material

cm2c03662_si_001.pdf

cm2c03662_si_002.cif

cm2c03662_si_003.cif

cm2c03662_si_004.cif

cm2c03662_si_005.cif

cm2c03662_si_006.cif

cm2c03662_si_007.cif

cm2c03662_si_008.cif

## References

[ref1] Uemura K.; Matsuda R.; Kitagawa S. Flexible Microporous Coordination Polymers. J. Solid State Chem. 2005, 178, 2420–2429. 10.1016/j.jssc.2005.05.036.

[ref2] Horike S.; Shimomura S.; Kitagawa S. Soft Porous Crystals. Nat. Chem. 2009, 1, 695–704. 10.1038/nchem.444. 21124356

[ref3] Férey G.; Serre C. Large Breathing Effects in Three-Dimensional Porous Hybrid Matter: Facts, Analyses, Rules and Consequences. Chem. Soc. Rev. 2009, 38, 1380–1399. 10.1039/b804302g. 19384443

[ref4] Schneemann A.; Bon V.; Schwedler I.; Senkovska I.; Kaskel S.; Fischer R. A. Flexible Metal-Organic Frameworks. Chem. Soc. Rev. 2014, 43, 6062–6096. 10.1039/C4CS00101J. 24875583

[ref5] Behera N.; Duan J.; Jin W.; Kitagawa S. The Chemistry and Applications of Flexible Porous Coordination Polymers. EnergyChem 2021, 3, 100067 10.1016/j.enchem.2021.100067.

[ref6] Wang S. Q.; Mukherjee S.; Zaworotko M. J. Spiers Memorial Lecture: Coordination Networks That Switch between Nonporous and Porous Structures: An Emerging Class of Soft Porous Crystals. Faraday Discuss. 2021, 231, 9–50. 10.1039/D1FD00037C. 34318839

[ref7] Forrest K. A.; Verma G.; Ye Y.; Ren J.; Ma S.; Pham T.; Space B. Methane Storage in Flexible and Dynamical Metal–Organic Frameworks. Chem. Phys. Rev. 2022, 3, 021308 10.1063/5.0072805.

[ref8] Nugent P.; Giannopoulou E. G.; Burd S. D.; Elemento O.; Giannopoulou E. G.; Forrest K.; Pham T.; Ma S.; Space B.; Wojtas L.; Eddaoudi M.; Zaworotko M. J. Porous Materials with Optimal Adsorption Thermodynamics and Kinetics for CO_2_ Separation. Nature 2013, 495, 80–84. 10.1038/nature11893. 23446349

[ref9] Fu M.; Wang Y.; Wang X.; Sun D. Metal-Organic Framework Materials for Light Hydrocarbon Separation. ChemPlusChem 2021, 86, 387–395. 10.1002/cplu.202000804. 33645928

[ref10] Cui X.; Chen K.; Xing H.; Yang Q.; Krishna R.; Bao Z.; Wu H.; Zhou W.; Dong X.; Han Y.; Li B.; Ren Q.; Zaworotko M. J.; Chen B. Pore Chemistry and Size Control in Hybrid Porous Materials for Acetylene Capture from Ethylene. Science 2016, 353, 141–144. 10.1126/science.aaf2458. 27198674

[ref11] Liang B.; Zhang X.; Xie Y.; Lin R. B.; Krishna R.; Cui H.; Li Z.; Shi Y.; Wu H.; Zhou W.; Chen B. An Ultramicroporous Metal-Organic Framework for High Sieving Separation of Propylene from Propane. J. Am. Chem. Soc. 2020, 142, 17795–17801. 10.1021/jacs.0c09466. 32991159 PMC10493866

[ref12] Bloch E. D.; Queen W. L.; Krishna R.; Zadrozny J. M.; Brown C. M.; Long J. R. Hydrocarbon Separations in a Metal-Organic Framework with Open Iron(II) Coordination Sites. Science 2012, 335, 1606–1610. 10.1126/science.1217544. 22461607

[ref13] He T.; Kong X. J.; Bian Z. X.; Zhang Y. Z.; Si G. R.; Xie L. H.; Wu X. Q.; Huang H.; Chang Z.; Bu X. H.; Zaworotko M. J.; Nie Z. R.; Li J. R. Trace Removal of Benzene Vapour Using Double-Walled Metal–Dipyrazolate Frameworks. Nat. Mater. 2022, 21, 689–695. 10.1038/s41563-022-01237-x. 35484330 PMC9156410

[ref14] Gu Z. Y.; Yan X. P. Metal-Organic Framework MIL-101 for High-Resolution Gaschromatographic Separation of Xylene Isomers and Ethlbenzene. Angew. Chem., Int. Ed. 2010, 49, 1477–1480. 10.1002/anie.200906560. 20091724

[ref15] Krause S.; Bon V.; Senkovska I.; Stoeck U.; Wallacher D.; Többens D. M.; Zander S.; Pillai R. S.; Maurin G.; Coudert F. X.; Kaskel S. A Pressure-Amplifying Framework Material with Negative Gas Adsorption Transitions. Nature 2016, 532, 348–352. 10.1038/nature17430. 27049950

[ref16] Taylor M. K.; Runčevski T.; Oktawiec J.; Gonzalez M. I.; Siegelman R. L.; Mason J. A.; Ye J.; Brown C. M.; Long J. R. Tuning the Adsorption-Induced Phase Change in the Flexible Metal-Organic Framework Co(Bdp). J. Am. Chem. Soc. 2016, 138, 15019–15026. 10.1021/jacs.6b09155. 27804295

[ref17] Wang S. Q.; Mukherjee S.; Patyk-Kaźmierczak E.; Darwish S.; Bajpai A.; Yang Q. Y.; Zaworotko M. J. Highly Selective, High-Capacity Separation of o-Xylene from C8 Aromatics by a Switching Adsorbent Layered Material. Angew. Chem. 2019, 131, 6702–6706. 10.1002/ange.201901198. 30791187

[ref18] Kundu T.; Wahiduzzaman M.; Shah B. B.; Maurin G.; Zhao D. Solvent-Induced Control over Breathing Behavior in Flexible Metal–Organic Frameworks for Natural-Gas Delivery. Angew. Chem. 2019, 131, 8157–8161. 10.1002/ange.201902738. 30913352

[ref19] Hazra A.; Van Heerden D. P.; Sanyal S.; Lama P.; Esterhuysen C.; Barbour L. J. CO2-Induced Single-Crystal to Single-Crystal Transformations of an Interpenetrated Flexible MOF Explained by in Situ Crystallographic Analysis and Molecular Modeling. Chem. Sci. 2019, 10, 10018–10024. 10.1039/C9SC04043A. 32015814 PMC6977545

[ref20] Ghosh S. K.; Zhang J. P.; Kitagawa S. Reversible Topochemical Transformation of a Soft Crystal of a Coordination Polymer. Angew. Chem., Int. Ed. 2007, 46, 7965–7968. 10.1002/anie.200703086. 17868167

[ref21] Bon V.; Klein N.; Senkovska I.; Heerwig A.; Getzschmann J.; Wallacher D.; Zizak I.; Brzhezinskaya M.; Mueller U.; Kaskel S. Exceptional Adsorption-Induced Cluster and Network Deformation in the Flexible Metal-Organic Framework DUT-8(Ni) Observed by in Situ X-Ray Diffraction and EXAFS. Phys. Chem. Chem. Phys. 2015, 17, 17471–17479. 10.1039/C5CP02180D. 26079102

[ref22] Loiseau T.; Serre C.; Huguenard C.; Fink G.; Taulelle F.; Henry M.; Bataille T.; Férey G. A Rationale for the Large Breathing of the Porous Aluminum Terephthalate (MIL-53) Upon Hydration. Chem. - Eur. J. 2004, 10, 1373–1382. 10.1002/chem.200305413. 15034882

[ref23] Mason J. A.; Oktawiec J.; Taylor M. K.; Hudson M. R.; Rodriguez J.; Bachman J. E.; Gonzalez M. I.; Cervellino A.; Guagliardi A.; Brown C. M.; Llewellyn P. L.; Masciocchi N.; Long J. R. Methane Storage in Flexible Metal-Organic Frameworks with Intrinsic Thermal Management. Nature 2015, 527, 357–361. 10.1038/nature15732. 26503057

[ref24] Steinfink H.; Brunton G. D. Crystal structure of erbium oxalate trihydrate. Inorg. Chem. 1970, 9, 2112–2115. 10.1021/ic50091a030.

[ref25] Gable R. W.; Hoskins B. F.; Robson R. A New Type of Interpenetration Involving Enmeshed Independent Square Grid Sheets. The Structure of Diaquabis-(4,4′-Bipyridine)Zinc Hexafluorosilicate. J. Chem. Soc. Chem. Commun. 1990, 23, 1677–1678. 10.1039/C39900001677.

[ref26] Fujita M.; Washizu S.; Ogura K.; Kwon Y. J. Preparation, Clathration Ability, and Catalysis of a Two-Dimensional Square Network Material Composed of Cadmium(II) and 4, 4′-Bipyridine. J. Am. Chem. Soc. 1994, 116, 1151–1152. 10.1021/ja00082a055.

[ref27] O’Hearn D. J.; Bajpai A.; Zaworotko M. J. The “Chemistree” of Porous Coordination Networks: Taxonomic Classification of Porous Solids to Guide Crystal Engineering Studies. Small 2021, 17, 2006351 10.1002/smll.202006351. 33690978

[ref28] Biradha K.; Mondai A.; Moulton B.; Zaworotko M. J. Coexisting Covalent and Non-Covalent Planar Networks in the Crystal Structures of {[M(Bipy)_2_(NO_3_)_2_]-Arene} (M = Ni, 1; Co, 2; Arène = Chlorobenzene, o-Dichlorobenzene, Benzene, Nitrobenzene, Toluene or Anisole)F. J. Chem. Soc., Daltan Trans. 2000, 2, 3837–3844. 10.1039/b003733h.

[ref29] Noro S. I.; Kitaura R.; Kondo M.; Kitagawa S.; Ishii T.; Matsuzaka H.; Yamashita M. Framework Engineering by Anions and Porous Functionalities of Cu(II)/4,4′-Bpy Coordination Polymers. J. Am. Chem. Soc. 2002, 124, 2568–2583. 10.1021/ja0113192. 11890808

[ref30] Kondo A.; Noguchi H.; Carlucci L.; Proserpio D. M.; Ciani G.; Kajiro H.; Ohba T.; Kanoh H.; Kaneko K. Double– step gas sorption of a two–dimensional metal–organic framework. J. Am. Chem. Soc. 2007, 129, 12362–12363. 10.1021/ja073568h. 17887754

[ref31] Blatov V. A.; Shevchenko A. P.; Proserpio D. M. Applied Topological Analysis of Crystal Structures with the Program Package Topospro. Cryst. Growth Des. 2014, 14, 3576–3586. 10.1021/cg500498k.

[ref32] Groom C. R.; Bruno I. J.; Lightfoot M. P.; Ward S. C. The Cambridge Structural Database. Acta Crystallogr., Sect. B: Struct. Sci., Cryst. Eng. Mater. 2016, 72, 171–179. 10.1107/S2052520616003954. PMC4822653 27048719

[ref33] Kumar N.; Wang S. Q.; Mukherjee S.; Bezrukov A. A.; Patyk-Kaźmierczak E.; O’Nolan D.; Kumar A.; Yu M. H.; Chang Z.; Bu X. H.; Zaworotko M. J. Crystal Engineering of a Rectangular Sql Coordination Network to Enable Xylenes Selectivity over Ethylbenzene. Chem. Sci. 2020, 11, 6889–6895. 10.1039/D0SC02123G. 33033602 PMC7500086

[ref34] Li D.; Kaneko K. Hydrogen Bond-Regulated Microporous Nature of Copper Complex-Assembled Microcrystals. Chem. Phys. Lett. 2001, 335, 50–56. 10.1016/S0009-2614(00)01419-6.

[ref35] Bon V.; Senkovska I.; Wallacher D.; Heerwig A.; Klein N.; Zizak I.; Feyerherm R.; Dudzik E.; Kaskel S. In Situ Monitoring of Structural Changes during the Adsorption on Flexible Porous Coordination Polymers by X-Ray Powder Diffraction: Instrumentation and Experimental Results. Microporous Mesoporous Mater. 2014, 188, 190–195. 10.1016/j.micromeso.2013.12.024.

[ref36] Hiraide S.; Tanaka H.; Ishikawa N.; Miyahara M. T. Intrinsic Thermal Management Capabilities of Flexible Metal-Organic Frameworks for Carbon Dioxide Separation and Capture. ACS Appl. Mater. Interfaces 2017, 9, 41066–41077. 10.1021/acsami.7b13771. 29068227

[ref37] Kanoh H.; Kondo A.; Noguchi H.; Kajiro H.; Tohdoh A.; Hattori Y.; Xu W. C.; Inoue M.; Sugiura T.; Morita K.; Tanaka H.; Ohba T.; Kaneko K. Elastic Layer-Structured Metal Organic Frameworks (ELMs). J. Colloid Interface Sci. 2009, 334, 1–7. 10.1016/j.jcis.2009.03.020. 19383559

[ref38] Ichikawa M.; Kondo A.; Noguchi H.; Kojima N.; Ohba T.; Kajiro H.; Hattori Y.; Kanoh H. Double-Step Gate Phenomenon in CO2 Sorption of an Elastic Layer-Structured MOF. Langmuir 2016, 32, 9722–9726. 10.1021/acs.langmuir.6b02551. 27599535

[ref39] Park J.; Sun L. B.; Chen Y. P.; Perry Z.; Zhou H. C. Azobenzene-Functionalized Metal-Organic Polyhedra for the Optically Responsive Capture and Release of Guest Molecules. Angew. Chem. 2014, 126, 5952–5956. 10.1002/ange.201310211. 24803325

[ref40] Baroncini M.; D’Agostino S.; Bergamini G.; Ceroni P.; Comotti A.; Sozzani P.; Bassanetti I.; Grepioni F.; Hernandez T. M.; Silvi S.; Venturi M.; Credi A. Photoinduced Reversible Switching of Porosity in Molecular Crystals Based on Star-Shaped Azobenzene Tetramers. Nat. Chem. 2015, 7, 634–640. 10.1038/nchem.2304. 26201739

[ref41] Lyndon R.; Konstas K.; Ladewig B. P.; Southon P. D.; Kepert P. C. J.; Hill M. R. Dynamic Photo-Switching in Metal-Organic Frameworks as a Route to Low-Energy Carbon Dioxide Capture and Release. Angew. Chem., Int. Ed. 2013, 52, 3695–3698. 10.1002/anie.201206359. 23401101

[ref42] Li B. A Novel Metal-Organic Framework as a Heterogeneous Catalysis for the Solvent-Free Conversion of CO_2_ and Epoxides into Cyclic Carbonate. Inorg. Chem. Commun. 2018, 88, 56–59. 10.1016/j.inoche.2017.12.014.

[ref43] He H.; Du J.; Su H.; Yuan Y.; Song Y.; Sun F. Four New Metal-Organic Frameworks Based on Bi-, Tetra-, Penta-, and Hexa-Nuclear Clusters Derived from 5-(Phenyldiazenyl)Isophthalic Acid: Syntheses, Structures and Properties. CrystEngComm 2015, 17, 1201–1209. 10.1039/C4CE01837K.

[ref44] Geng K.; Yang X.; Zhao Y.; Cui Y.; Ding J.; Hou H. Efficient Strategy for Investigating the Third-Order Nonlinear Optical (NLO) Properties of Solid-State Coordination Polymers. Inorg. Chem. 2022, 61, 12386–12395. 10.1021/acs.inorgchem.2c01785. 35895943

[ref45] Halder G. J.; Kepert C. J.; Moubaraki B.; Murray K. S.; Cashion J. D. Guest-Dependent Spin Crossover in a Nanoporous Molecular Framework Material. Science 2002, 298, 1762–1765. 10.1126/science.1075948. 12459583

[ref46] Gardner G. B.; Venkataraman D.; Moore J. S.; Lee S. Spontaneous assembly of a hinged coordination network. Nature 1995, 374, 792–795. 10.1038/374792a0.

[ref47] Barbour L. J. X-Seed 4: Updates to a Program for Small-Molecule Supramolecular Crystallography. J. Appl. Crystallogr. 2020, 53, 1141–1146. 10.1107/S1600576720007438.

[ref48] Li Y.; Gai T.; Lin Y.; Zhang W.; Li K.; Liu Y.; Duan Y.; Li B.; Ding J.; Li J. Eight Cd(Ii) Coordination Polymers with Persistent Room-Temperature Phosphorescence: Intriguing Dual Emission and Time-Resolved Afterglow Modulation. Inorg. Chem. Front. 2020, 7, 777–785. 10.1039/C9QI01273G.

[ref49] Dias I. M.; Junior H. C. S.; Costa S. C.; Cardoso C. M.; Cruz A. G. B.; Santos C. E. R.; Candela D. R. S.; Soriano S.; Marques M. M.; Ferreira G. B.; Guedes G. P. Mononuclear Coordination Compounds Containing a Pyrazole-Based Ligand: Syntheses, Magnetism and Acetylcholinesterase Inhibition Assays. J. Mol. Struct. 2020, 1205, 127564 10.1016/j.molstruc.2019.127564.

[ref50] Morley F. V. Linkages. Sci. Mon. 1919, 9, 366–378.

[ref51] Kondo A.; Chinen A.; Kajiro H.; Nakagawa T.; Kato K.; Takata M.; Hattori Y.; Okino F.; Ohba T.; Kaneko K.; Kanoh H. Metal-Ion-Dependent Gas Sorptivity of Elastic Layer-Structured MOFs. Chem. - Eur. J. 2009, 15, 7549–7553. 10.1002/chem.200901208. 19569143

[ref52] Bon V.; Kavoosi N.; Senkovska I.; Kaskel S. Tolerance of Flexible MOFs toward Repeated Adsorption Stress. ACS Appl. Mater. Interfaces 2015, 7, 22292–22300. 10.1021/acsami.5b05456. 26397165

[ref53] Li L.; Krishna R.; Wang Y.; Wang X.; Yang J.; Li J. Flexible Metal–Organic Frameworks with Discriminatory Gate-Opening Effect for the Separation of Acetylene from Ethylene/Acetylene Mixtures. Eur. J. Inorg. Chem. 2016, 2016, 4457–4462. 10.1002/ejic.201600182.

[ref54] Hyunho K. K.; Yang S.; Narayanan S.; Kapustin E. A.; Furukawa H.; Umans A. S.; Yaghi O. M.; Wang E. N. Powered By Natural Sunlight. Science 2017, 434, 430–434. 10.1126/science.aam8743 28408720

[ref55] Towsif Abtab S. M.; Alezi D.; Bhatt P. M.; Shkurenko A.; Belmabkhout Y.; Aggarwal H.; Weseliński ŁJ.; Alsadun N.; Samin U.; Hedhili M. N.; Eddaoudi M. Reticular Chemistry in Action: A Hydrolytically Stable MOF Capturing Twice Its Weight in Adsorbed Water. Chem 2018, 4, 94–105. 10.1016/j.chempr.2017.11.005.

[ref56] Arundel A. V.; Sterling E. M.; Biggin J. H.; Sterling T. D. Indirect Health Effects of Relative Humidity in Indoor Environments. Environ. Health Perspect. 1986, 65, 351–361. 10.1289/ehp.8665351. 3709462 PMC1474709

[ref57] Llewellyn P. L.; Schüth F.; Grillet Y.; Rouquerol F.; Rouquerol J.; Unger K. K. Water sorption on mesoporous aluminosilicate MCM-41. Langmuir 1995, 11, 574–577. 10.1021/la00002a036.

[ref58] Abdulhalim R. G.; Bhatt P. M.; Belmabkhout Y.; Shkurenko A.; Adil K.; Barbour L. J.; Eddaoudi M. A Fine-Tuned Metal-Organic Framework for Autonomous Indoor Moisture Control. J. Am. Chem. Soc. 2017, 139, 10715–10722. 10.1021/jacs.7b04132. 28661666

[ref59] Zhu N. X.; Wei Z. W.; Chen C. X.; Xiong X. H.; Xiong Y. Y.; Zeng Z.; Wang W.; Jiang J. J.; Fan Y. N.; Su C. Y. High Water Adsorption MOFs with Optimized Pore-Nanospaces for Autonomous Indoor Humidity Control and Pollutants Removal. Angew. Chem., Int. Ed. 2022, 61, e202112097 10.1002/anie.202112097. 34779556

