# Supplementary material for: Structural Phase Transformations Induced by Guest Molecules in a Nickel-Based 2D Square Lattice Coordination Network
Source: Chem Mater. 2023 Jan 10;35(2):783–91. doi: 10.1021/acs.chemmater.2c03662 (PMC9878710; doi:10.1021/acs.chemmater.2c03662)
Supplement: Supplementary file 1 — cm2c03662_si_001.pdf [file cm2c03662_si_001.pdf]

Supporting Information

Structural Phase Transformations Induced by Guest Molecules in a Nickel-based 2D Square Lattice Coordination Network

Xia Li,† Debobroto Sensharma,† Varvara I. Nikolayenko,† Shaza Darwish,† Andrey A. Bezrukov, † Naveen Kumar,† Wansheng Liu,‡ Xiang-Jing Kong,† Zhenjie Zhang,‡ Michael J. Zaworotko† *

†Department of Chemical Science, Bernal Institute, University of Limerick, Limerick, V94 T9PX, Republic of Ireland

‡College of Chemistry, Nankai University, Tianjin 300071, People's Republic of China

Table of Contents

1. Experimental Section S2
2. Characterization Section S2-S4
3. Survey of Crystallographic and Databases S5-S6
4. General Characterization Data S7-S17
5. Cycling DVS Data S18
6. FT-IR Data S19
7. Database Survey of azpy Based **sql** Networks S20-S21
8. Crystallographic Data and Structural Analysis S22-S28
9. Database Survey of FMOMs Induced by Water S29-S30
10. References S31-S33

1. Survey of crystallographic and topological databases

The list of MOMs having **sql** net topology was obtained from the TTO TOPOS database[1] (version: December 2021); valence-bonded MOFs in standard representation were used. The enlisted MOM crystal structures from the TTO database were analyzed using queries to the Cambridge Structural Database (CSD version 5.43, November 2022) through the CSD Application Programming Interface (CSD Python API).[2] **Sql** networks with interlayer Hydrogen bonds were identified using custom-written Python script which implements the algorithm illustrated in Figure S1.

2. Materials and Synthesis

All chemicals dimethyl 5-aminoisophthalate, nitrosobenzene and 4-aminopyridine were obtained commercially and used as received without further purification. Synthesis of (*E*)-1,2-di(pyridin-4-yl)diazene (azpy) and (*E*)-5-(phenyldiazenyl)isophthalic acid (H_2pdia) were accomplished by using previously reported procedures.[3,4]

3. Single-crystal X-ray diffraction measurements.

Single-crystal reflection data were collected on a Bruker D8 Quest diffractometer equipped with a Photon 100 detector and a Cu K_α microfocus source (λ = 1.5406 Å). Diffraction images were collected in shutterless mode, and were indexed, integrated and scaled in APEX3.[5] By using the multi-scan method SADABS, absorption correction was performed.[6] Space group determination was performed with structure solution using SHELXT intrinsic phasing and the solution was refined on F2 using SHELXL non-linear least squares implemented in Olex2 v1.2.10.[7,8] All non-hydrogen framework atoms were refined with anisotropic parameters, while H atoms were placed in calculated positions and refined by using a riding model. All the crystals were measured under liquid N_2 flow at temperature of 100K or 150K to avoid the phase transformation caused by escape of guest molecules. Crystallographic data and structural refinement information are listed in Tables S1. All the structure of six phases (**sql-(azpy)(pdia)-Ni-α_{DMF}, β and α_{H2O}, α_{DCM}, α_{PX}, α_{EB}**) were solved and refined in *Pc* space group. Crystallographic data for the structures reported in this paper have been deposited with the Cambridge Crystallographic Data Centre as supplementary publication no. CCDC 2178495 – 2178499, 2212543, 2224805. The torsion angle in Figure 2(c) was measured by superposing the isophthalate ring for (E)-5-(phenyldiazenyl) isophthalic acid and measuring the torsion angles of -C_{20}-C_{19}-N_5-N_6- in **α_{DMF}** (5.39 °) and -C_{20}-C_{19}-N_5-N_6- in **β** (38.09 °). In Figure 2 (d), the torsion angle was measured by superposing one pyridyl ring of (E)-1,2-di(pyridin-4-yl)diazene and measuring the -C_7-C_6-N_3-N_2- torsion angle in **α_{DMF}** (11.71 °) and the -C_7-C_6-N_3-N_2- torsion angle in **β** (-37.28 °).

4. IR spectra Fourier Transform Infrared (FTIR) Spectroscopy

Spectra were obtained by using a FTIR spectrometer (Agilent technologies, Cary 630) in the range of wavelength 4000-650 cm^{-1}.

5. Thermogravimetric analysis (TGA)

Thermogravimetric analyses (TGA) were performed under N_2 using a TA Instruments Q50 system. Samples were loaded into aluminum sample pans and heated at rate of 10 °C·min^{-1} from room temperature to 500 °C.

6. Powder X-ray diffraction measurements

Powder X-ray diffraction patterns were recorded on a PANalytical X'Pert MPD Pro (Cu K_α, λ = 1.5418 Å) with a 1D X'Celerator strip detector. Experiments were conducted in continuous scanning mode with the goniometer in the theta-theta orientation. Incident beam optics included the Fixed Divergences slit with anti-scatter slit PreFIX module, with a 1/8°divergence slit and a 1/4° anti-scatter slit, as well as a 10 mm fixed incident beam mask and a Soller slit (0.04 rad). Divergent beam optics included a P7.5 anti-scatter slit, a Soller slit (0.04 rad), and a Ni β filter. The data were collected in the range of 2θ = 3-40°. Raw data was then evaluated using the X'Pert HighScore Plus™ software V 4.1 (PANalytical, The Netherlands).

7. Variable Temperature Powder X-ray Diffraction (VT-PXRD)

Diffractograms at different temperature were recorded using a PANalytical X'Pert Pro-MPD diffractometer equipped with a PIXcel3D detector operating in scanning line detector mode with an active length of 4 utilizing 255 channels. Anton Paar TTK 450 stage coupled with the Anton Paar TCU 110 Temperature Control Unit was used to record the variable temperature diffractograms. The diffractometer is outfitted with an Empyrean Cu LFF (long fine focus) HR (9430 033 7300x) tube operated at 40 kV and 40 mA and Cu K_α radiation (λ_α = 1.54056 Å) was used for diffraction experiments. Use continuous scanning mode with the goniometer in the theta-theta orientation to collect the data. Incident beam optics included the Fixed Divergences slit, with a 1/4° divergence slit and a Soller slit (0.04 rad). Divergent beam optics included a P7.5 anti-scatter slit, a Soller slit (0.04 rad), and a Ni-β filter. In a typical experiment, 20 mg of sample was ground into a fine powder and was loaded on a zero-background sample holder made for Anton Paar TTK 450 chamber. The data was collected from 4°- 40° (2θ) with a step-size of 0.0167113° and a scan time of 50 seconds per step. Crude data were analyzed using the X'Pert HighScore Plus™ software V 4.1 (PANalytical, The Netherlands). Sample was heated up to 473 K.

Humidity swing PXRD test was conducted on PANalytical X'Pert, the dry test condition was achieved by apply dry N_2 flow with 40 min/L for 10 min, and humid test condition was achieved by cutting off N_2 flow first and put 2mL of liquid water in the closed chamber for 1h.

8. Gas adsorption measurements.

For gas sorption experiments, high-purity gases were used as received from BOC Gases Ireland: He (99.999%), N_2 (99.9992%), CO_2 (99.995%). Low pressure (0-1 bar) CO_2 and N_2 sorption isotherms were measured using Micromeritics 3flex instrument. Methanol (MeOH) exchanged **sql-(azpy)(pdia)-Ni** was degassed under high vacuum at 100 °C for 10 h on Micromeritics Smart VacPrep instrument to obtain **sql-(azpy)(pdia)-Ni-β**. The activated sample (100 mg) were transferred to 3Flex and evacuated at room temperature for 10 hours before the measurements. The temperature at 77 K and 195 K were maintained using a 4L Dewar filled with liquid nitrogen and a dry ice-acetone mixture respectively. Bath temperature of 273 K was precisely controlled with a Julabo ME (v.2) recirculating control system containing a mixture of ethylene glycol and water.

9. Kinetics and recyclability tests

The kinetics and recyclability tests were performed at 25 °C on Surface Measurement Systems DVS Intrinsic using air as a carrier gas to gravimetrically measure the uptake and loss of vapor. The mass

of the sample was determined by comparison to an empty reference pan and recorded by a high-resolution microbalance with a precision of 0.1 μg. Kinetics was measured between two points 0, 90% RH and 40%, 60% RH separately with a convergence equilibrium criterion dm/dt = 0.01 %/min. The recyclability test was done by performing 100 cycles, each cycle consisting of 10 min adsorption step (90% RH) and 10 min desorption step (0% RH) and 12 cycles between 40% (25 min, desorption) - 60% (60 min, adsorption) RH.

10. Stability test

sql-(azpy)(pdia)-Ni-β (100 mg, 0.19 mmol) was immersed in water or aqueous solutions at different pH values or else conditioned in an environmental chamber (relative humidity = 95 %; T = 45 °C). After 24 hours samples were filtered off, washed with water, and dried at ambient conditions for 30 min.

Supporting figures and tables

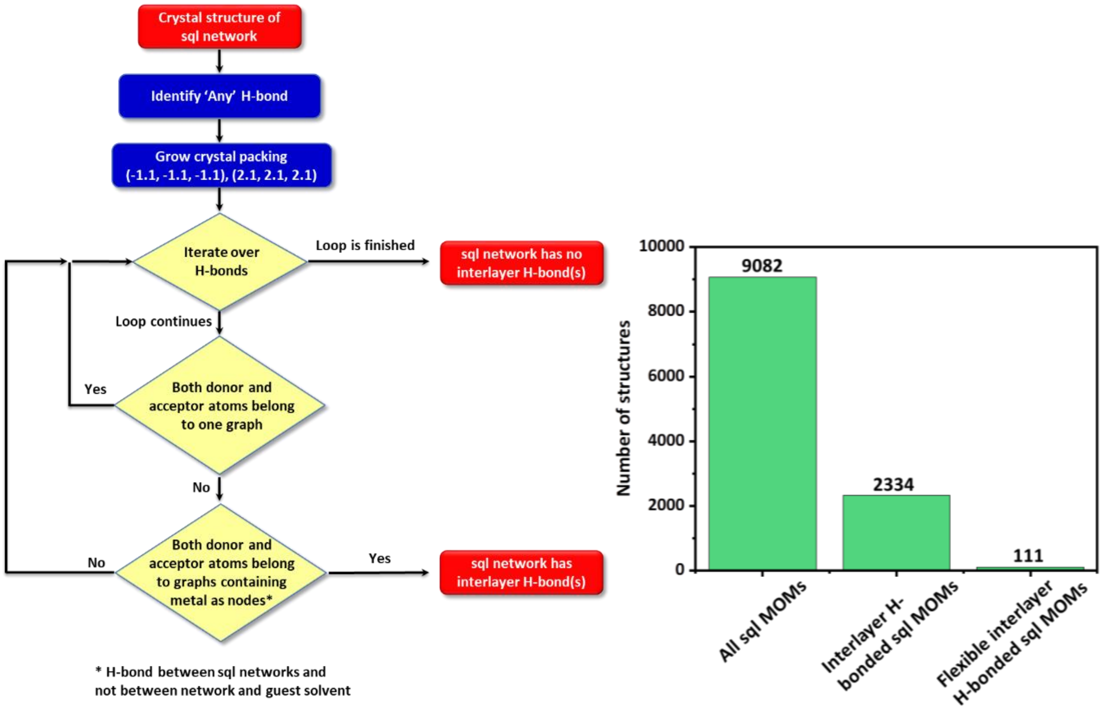

Figure S1. (Left) Flow chart of the algorithm used for identification of interlayer H-bonds in **sql** nets. (Right) Number of structures reported for different types of **sql** MOMs.

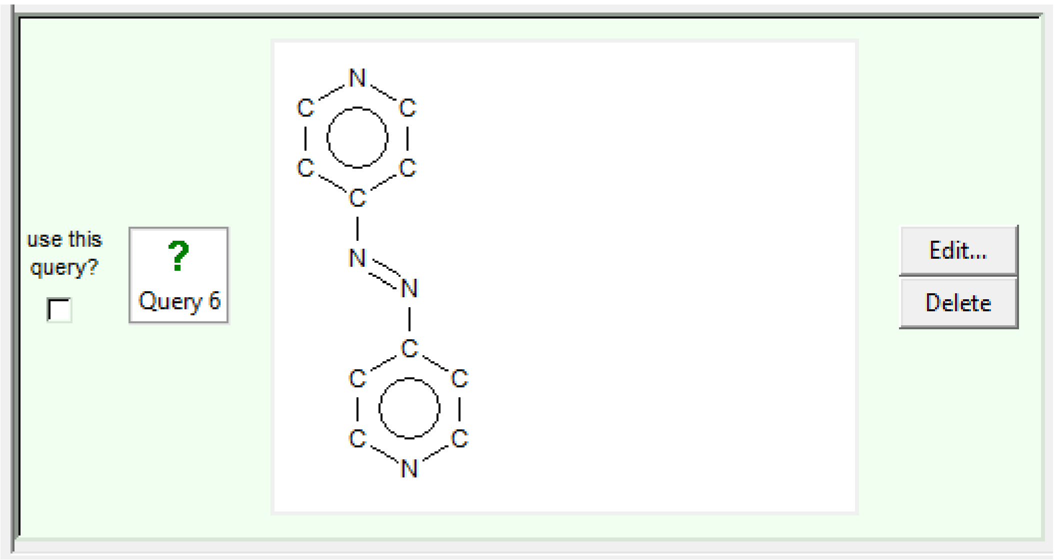

Figure S2. ConQuest request used to find sql networks based on azpy linker found in the TOPOS TTO ∩ CSD database, Table S1.

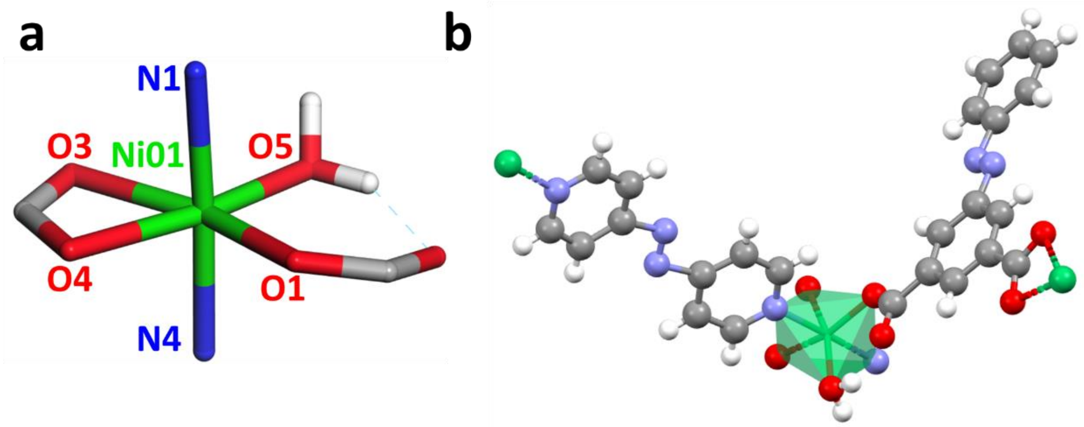

Figure S3. (a) MBB in **sql-(azpy)(pdia)-Ni**. (b) Two ligands and one water molecule connected with an octahedral environment of MBB.

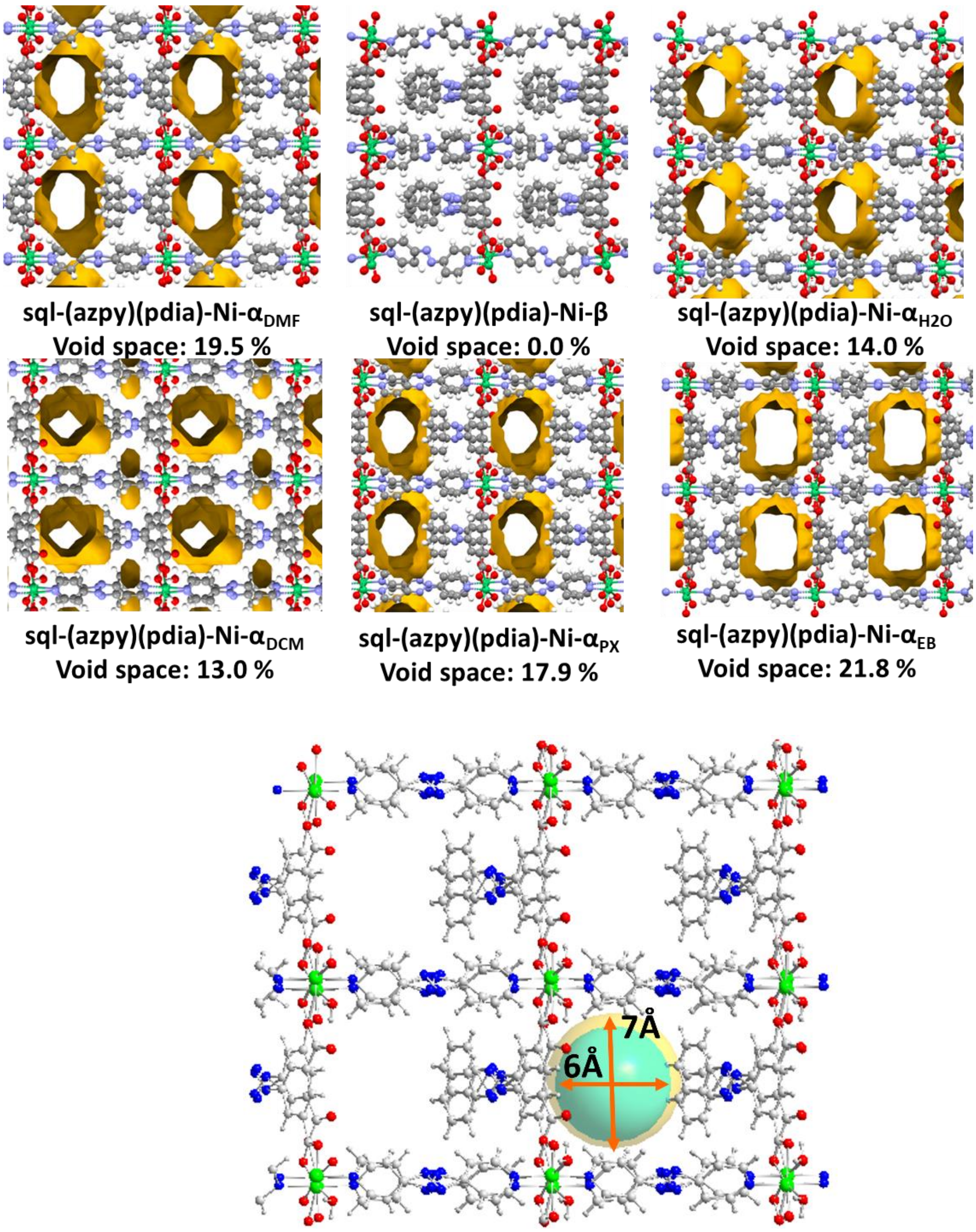

Figure S4. View along the *c* axis of the 2D frameworks and structures of **sql-(azpy)(pdia)-Ni-α$_{DMF}$**, **sql-(azpy)(pdia)-Ni-β** and **sql-(azpy)(pdia)-Ni-α$_{H2O}$**, **sql-(azpy)(pdia)-Ni-α$_{DCM}$**, **sql-(azpy)(pdia)-Ni-α$_{PX}$**, **sql-(azpy)(pdia)-Ni-α$_{EB}$**. Pore size of **sql-(azpy)(pdia)-Ni-α$_{DMF}$**. The void dimensions were measured with Mercury software after deleting guest molecules, using probe radius 1.2, approximate grid spacing 0.7 and the contact surface. The pore size was then calculated with the Diamond software package by inserting an atom into the middle of the channel (selecting two diagonal carbon atoms of the channel and inserting an atom between them) and making the atom edge tangent to the two sides of the pore walls in the channels. The diameters of the inserted atoms were then considered to represent the pore size of the channel.

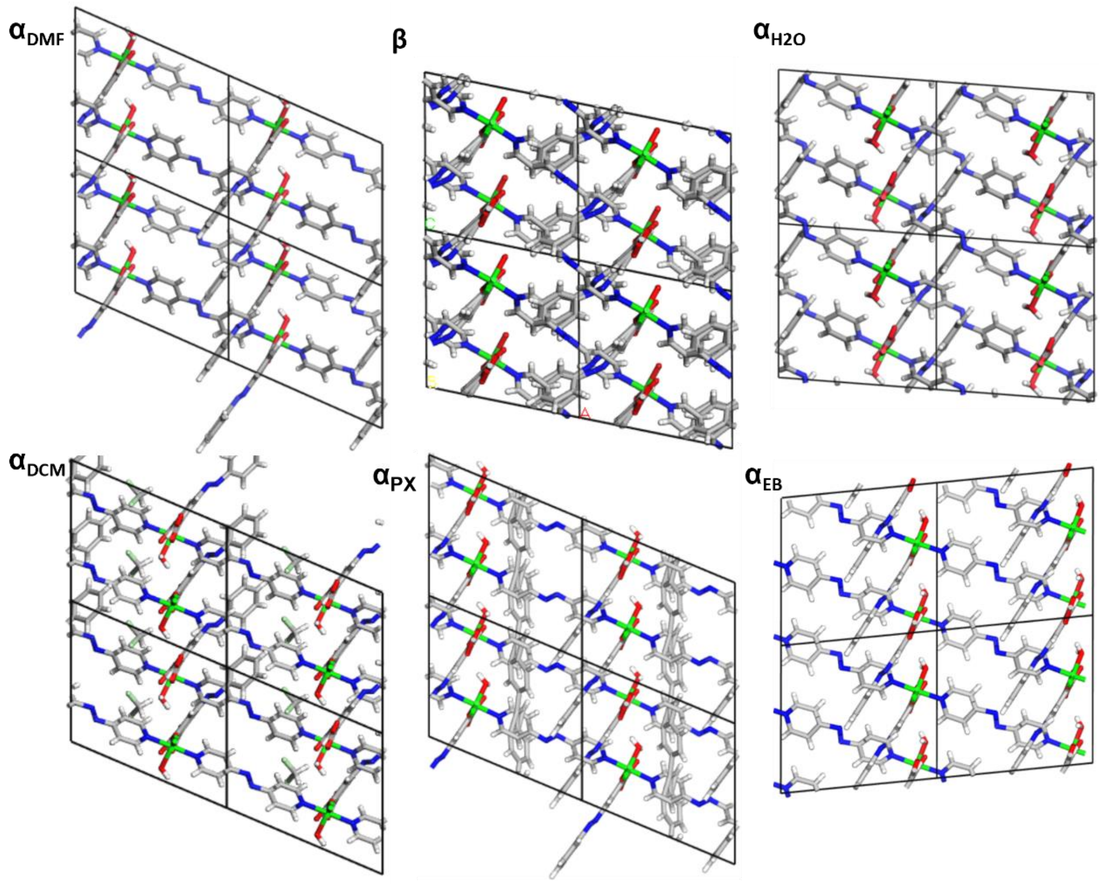

Figure S5. View along the *b* axis of the 2D frameworks and structures of **sql-(azpy)(pdia)-Ni-α_{DMF}**, **sql-(azpy)(pdia)-Ni-β** and **sql-(azpy)(pdia)-Ni-α_{H2O}**, **sql-(azpy)(pdia)-Ni-α_{DCM}**, **sql-(azpy)(pdia)-Ni-α_{PX}**, **sql-(azpy)(pdia)-Ni-α_{EB}**.

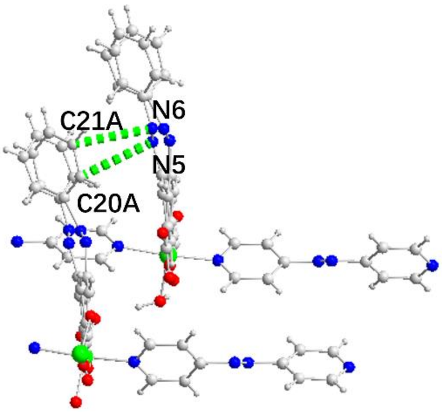

Figure S6. Close contacts in **sql-(azpy)(pdia)-Ni-β** ($d_{C21A \cdots N6}$ = 3.42 Å, $d_{C20A \cdots N5}$ = 3.43 Å).

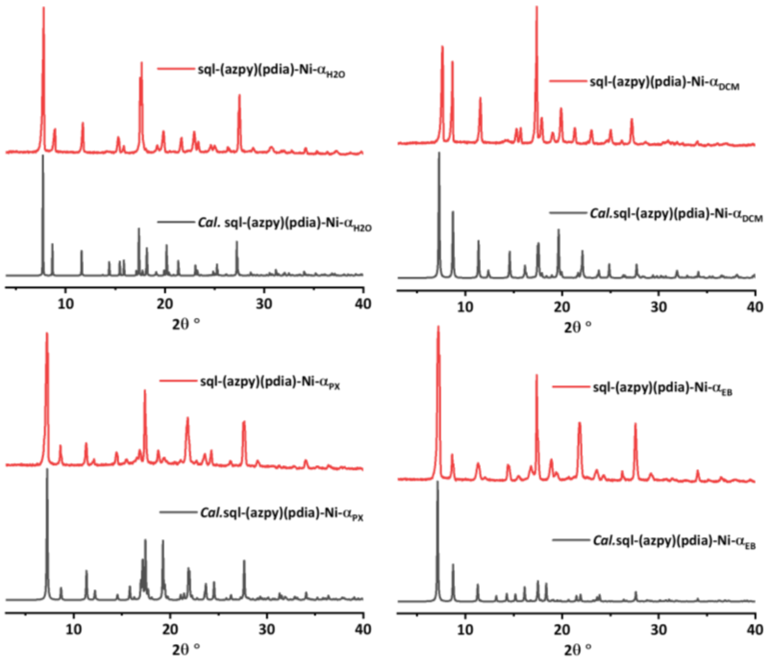

Figure S7. Calculated and experimental PXRD patterns of **sql-(azpy)(pdia)-Ni-α_{H2O}**, **sql-(azpy)(pdia)-Ni-α_{DCM}**, **sql-(azpy)(pdia)-Ni-α_{PX}** and **sql-(azpy)(pdia)-Ni-α_{EB}**.

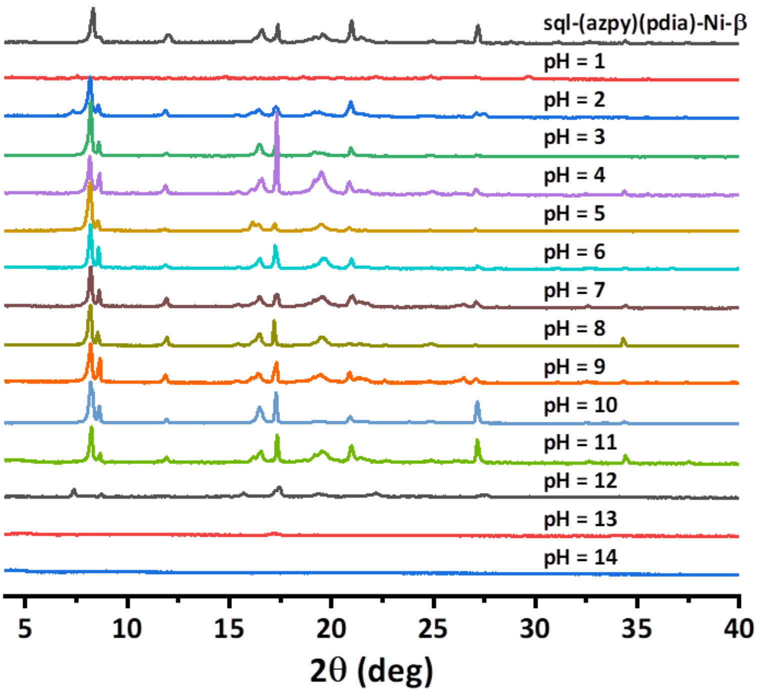

Figure S8. PXRD patterns of activated **sql-(azpy)(pdia)-Ni-β** (black) and **sql-(azpy)(pdia)-Ni-β** soaked in aqueous solution of pH = 1 to pH = 14.

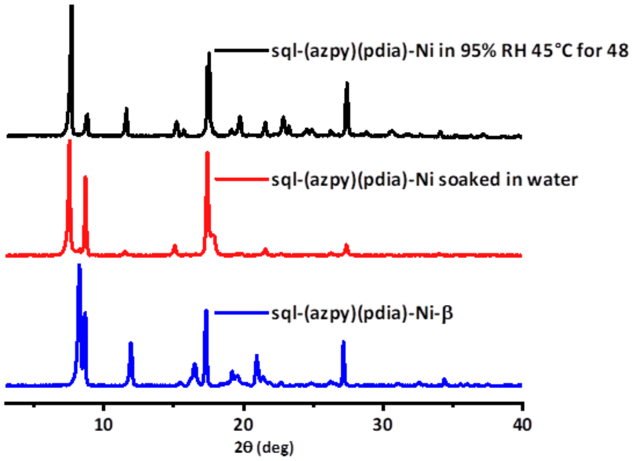

Figure S9. PXRD patterns of activated **sql-(azpy)(pdia)-Ni-β** (blue); **sql-(azpy)(pdia)-Ni-β** soaked in water to obtain **sql-(azpy)(pdia)-Ni-α_{H2O}** (red); **sql-(azpy)(pdia)-Ni-β** exposed in 95% RH at 45°C for 48h to obtain **sql-(azpy)(pdia)-Ni-α_{H2O}** (black).

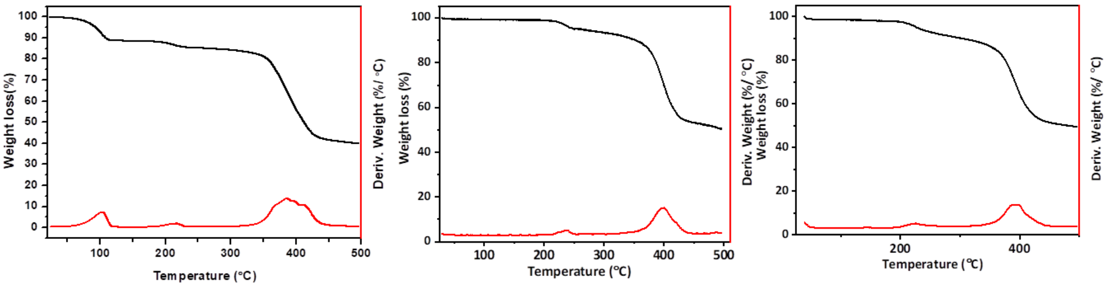

sql-(azpy)(pdia)-Ni-α_{DMF} **sql-(azpy)(pdia)-Ni-β** **sql-(azpy)(pdia)-Ni-α_{H2O}**

Figure S10. Thermogravimetric Analysis (TGA) of **sql-(azpy)(pdia)-Ni-α_{DMF}**, **sql-(azpy)(pdia)-Ni-β** and **sql-(azpy)(pdia)-Ni-α_{H2O}**.

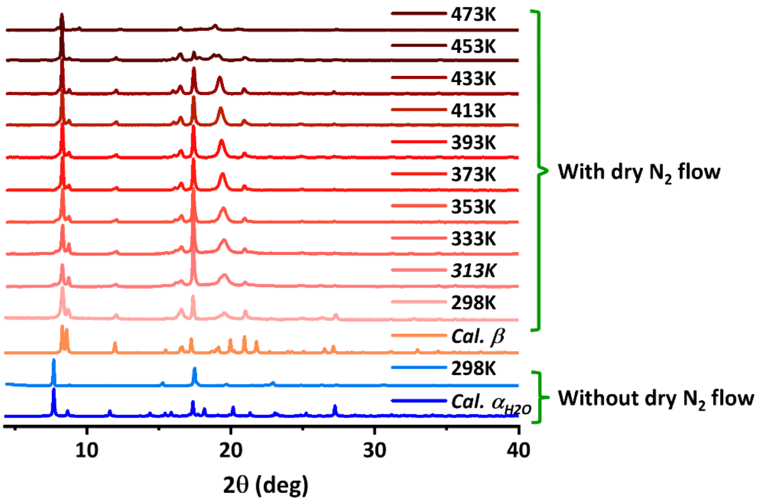

Figure S11. Variable temperature PXRD patterns conducted on **sql-(azpy)(pdia)-Ni-α_{H2O}**. First experimental PXRD pattern collected on sample which just took from liquid water at 298K without dry N_2 flow; After dry N_2 flow applied on sample, temperature increased from 298K to 473K.

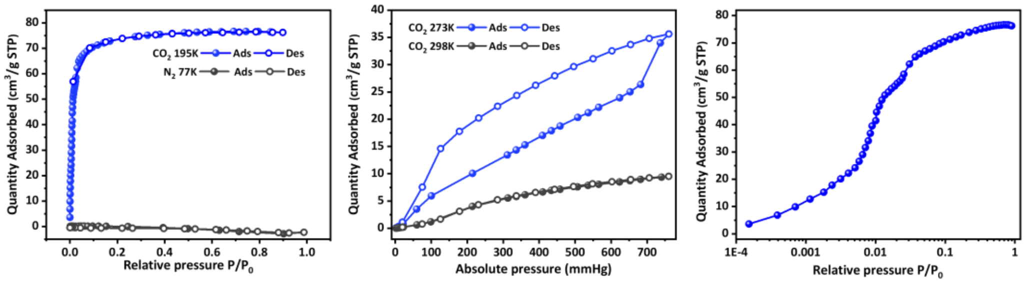

Figure S12. (Left) Sorption isotherms for **sql-(azpy)(pdia)-Ni** of CO_2 in 195 K (blue) and N_2 sorption isotherm in 77K (black). (Middle) Sorption isotherms for **sql-(azpy)(pdia)-Ni** of CO_2 in 273K (blue) and 298K (black). (Right) Log plot of CO_2 adsorption isotherm for **sql-(azpy)(pdia)-Ni** at 195 K.

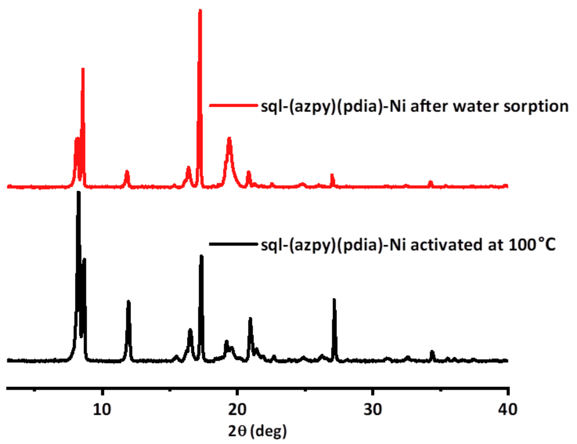

Figure S13. PXRD patterns of activated **sql-(azpy)(pdia)-Ni-β** (black), **sql-(azpy)(pdia)-Ni-β** after 100 cycles DVS tests (red).

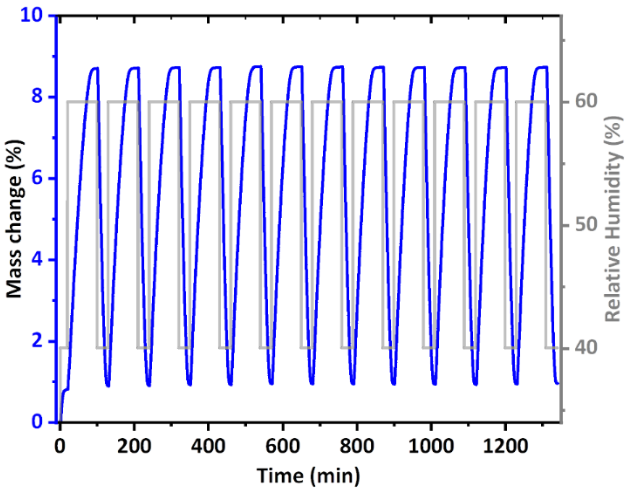

Figure S14. Cycling experiment showing dynamic water adsorption−desorption of **sql-(azpy)(pdia)-Ni** with 12 cycles between 40 – 60% RH on 11.2 mg of sample at 298 K.

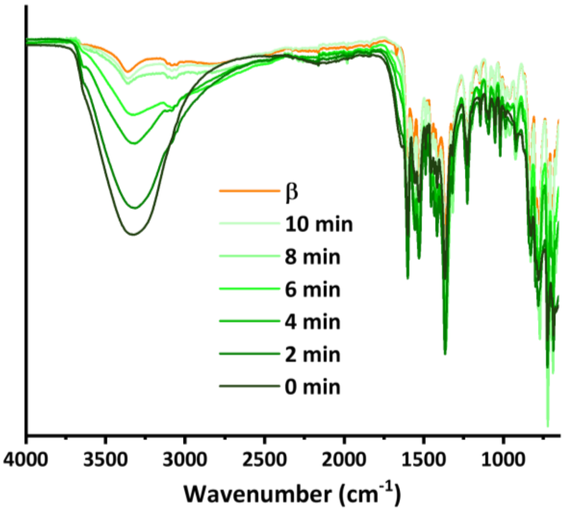

Figure S15. FT-IR spectra of **sql-(azpy)(pdia)-Ni**. First experimental FT-IR spectrum collected on sample which just took from liquid water at 293K (**sql-(azpy)(pdia)-Ni-α_{H2O}**), every 2 min collected one spectrum. From the results of O-H water stretching peak at 3326 cm^{-1} in the IR spectra, guest water in **sql-(azpy)(pdia)-Ni-α_{H2O}** gradually lost withnin 10 min (ambient condition: 20 °C, 40% RH), left peak in 3356 cm^{-1} at 10min and **β** due to coordinated water in **β** (3356 cm^{-1}).

Table S1. 55 **sql** networks based on azpy linker found in the TOPOS TTO∩CSD database survey.

Type of sql net	Second linker	RefCode	DOI reference
Single-linker	-	AKONIX	CCDC 147902
Single-linker	-	GUTDOO	10.1126/science.1075948
Single-linker	-	GUTDOO01	10.1126/science.1075948
Single-linker	-	GUTDOO02	10.1002/cplu.202000557
Single-linker	-	GUTDUU	10.1126/science.1075948
Single-linker	-	MAHYEZ	10.1039/a807924b
Single-linker	-	MAHYID	10.1039/a807924b
Single-linker	-	MUMNAM	10.1039/D0CE00142B
Single-linker	-	MUMNAM01	10.1039/D0CE00142B
Single-linker	-	NARXEK	10.1021/ic048371u
Single-linker	-	NARXIO	10.1021/ic048371u
Single-linker	-	QOTTUP	10.1524/ncrs.2009.0088
Single-linker	-	REBZIH	10.1021/cm990612m
Single-linker	-	RECLOA	10.1021/cm990612m
Single-linker	-	TANVEK	10.1071/CH04290
Single-linker	-	TANVOU	10.1071/CH04290
Single-linker	-	WOJKEN	10.1107/S1600536814016158
Single-linker	-	XEFGEU	10.1039/b006543i
Single-linker	-	XEFGEU01	10.1021/jacs.7b01682
Single-linker	-	XEFKUO	10.1002/1099-0682(200012)2000:12<2549:AID-EJIC2549>3.3.CO;2-4
Single-linker	-	XEFLAV	10.1002/1099-0682(200012)2000:12<2549::AID-EJIC2549>3.3.CO;2-4
Single-linker	-	XESBED	10.1021/ic061052d
Single-linker	-	XESBIH	10.1021/ic061052d
Single-linker	-	XETNUG	10.1021/ic061052d
Single-linker	-	XETPAO	10.1021/ic061052d
Single-linker	-	XETPES	10.1021/ic061052d
Mixed-linker	dicarboxylate	DADRIN	10.1002/chem.202001611
Mixed-linker	dicarboxylate	EHOVIH	10.1016/j.inoche.2010.05.018
Mixed-linker	dicarboxylate	IRORAK	10.1021/acs.cgd.6b00946
Mixed-linker	dicarboxylate	IRORIS	10.1021/acs.cgd.6b00946
Mixed-linker	dicarboxylate	JOYNET	10.1039/C9CE01719D
Mixed-linker	dicarboxylate	MUBXUD	10.1107/S1600536809030189
Mixed-linker	dicarboxylate	OGIHIW	10.1039/b907895a
Mixed-linker	dicarboxylate	OGIHIW01	10.1002/ejic.200901254

Mixed-linker	dicarboxylate	QIWBAB	10.1039/C3CE42454E
Mixed-linker	dicarboxylate	REHPOL	10.1039/c2ce25561h
Mixed-linker	dicarboxylate	REHPUR	10.1039/c2ce25561h
Mixed-linker	dicarboxylate	SAXRAM	10.1021/cg3000813
Mixed-linker	dicarboxylate	SAXROA	10.1021/cg3000813
Mixed-linker	dicarboxylate	SOXPON	CCDC 1843749
Mixed-linker	dicarboxylate	SUSQIH	10.1002/ejic.200901254
Mixed-linker	dicarboxylate	URABEW	10.1039/C6CE01173J
Mixed-linker	dicarboxylate	UVOSUV	10.1016/j.poly.2016.08.044
Mixed-linker	dicarboxylate	ZIKCIH	10.1039/C3CE40858B
Mixed-linker	N-donor-carboxylate	LURXAX	10.1002/cjoc.20020200214
Mixed-linker	N-donor-carboxylate	WIWXEH	10.1016/j.inoche.2013.12.015
Mixed-linker	N-donor-carboxylate	CUWVIA	10.1039/b915440j
Mixed-linker	N-donor-carboxylate	DOTFIE	10.1016/j.ica.2019.05.038
Mixed-linker	N-donor-carboxylate	LURXAX	CCDC 149036
Mixed-linker	N-donor-carboxylate	MEMFUI	10.21060/cis.2016.431
Mixed-linker	N-donor-carboxylate	SEKWOX	10.11862/CJIC.2017.189
Mixed-linker	N-donor	DAPMAK	10.1021/ic202129a
Mixed-linker	N-donor	POPFIM	10.11862/CJIC.2019.176
Mixed-linker	N-donor	SIDYUC	10.1002/anie.201802774
Single-linker	-	VUDBOO[a]	10.1039/C9TA09928J
Single-linker	-	XEBTAC[a]	10.1021/jacs.7b01682
Mixed-linker	dicarboxylate	CAQMOA[a]	10.1021/acs.inorgchem.1c02062
Mixed-linker	dicarboxylate	NOFRAD[a]	10.1039/C4CE00830H
Mixed-linker	dicarboxylate	BEHLIN[a]	10.1021/acs.inorgchem.2c01785

[a] *examples found in CSD database but outside of TOPOS TTO database.*

Table S2. Selected crystallographic data and structure refinement summary for **sql-(azpy)(pdia)-Ni.**

**Compounds**	**sql-(azpy)(pdia)-Ni-α_{DMF}**	**sql-(azpy)(pdia)-Ni-β**	**sql-(azpy)(pdia)-Ni-α_{H2O}**	**sql-(azpy)(pdia)-Ni-β'**
	(Open)	***(Close)***	***(Open)***	***(Close-Open-Close)***
Identification code	2178499	2224805	2178497	2212543
Empirical formula	$C_{27}H_{25}N_7O_6Ni$	$C_{24}H_{16}N_6O_5Ni$	$C_{24}H_{18}N_6O_7Ni$	$C_{24}H_{18}N_6NiO_5$
Formula weight	602.25	527.14	561.15	529.15
Temperature/K	150(2)	148.15	148(2)	100.00
Crystal system	monoclinic	monoclinic	monoclinic	monoclinic
Space group	*Pc*	*Pc*	*Pc*	*Pc*
a/Å	13.2072(4)	10.8795(6)	11.4324(3)	10.9088(11)
b/Å	10.1554(3)	10.2690(6)	10.2070(2)	10.2857(10)
c/Å	11.2001(4)	10.9631(6)	11.1539(3)	10.9669(11)
α /°	90	90	90	90
β /°	113.9200(10)	101.769(3)	95.0880(10)	101.854(6)
γ /°	90	90	90	90
Volume/$Å^3$	1373.19(8)	1199.07(12)	1296.43(5)	1204.3(2)
Z	2	2	2	2
ρ calcg/cm^3	1.457	1.46	1.438	1.459
μ /mm^{-1}	0.761	1.582	1.557	1.576
F(000)	624.0	540	576.0	544.0
Crystal size/mm^3	0.2 × 0.2 × 0.2	0.2 × 0.2 × 0.2	0.2 × 0.2 × 0.2	0.2 × 0.2 × 0.1
Radiation	MoKα (λ = 0.71073)	CuKα (λ = 1.54178)	CuKα (λ = 1.54178)	CuKα (λ = 1.54178)
2Θ range for data collection/°	6.75 to 56.672	8.61 to 149.212	11.642 to 144.36	8.282 to 140.066
Index ranges	-17 ⩽ h ⩽ 17, -13 ⩽ k ⩽ 13, -14 ⩽ l ⩽ 14	-11 ⩽ h ⩽ 13, -12 ⩽ k ⩽ 12, -13 ⩽ l ⩽ 10	-14 ⩽ h ⩽ 14, -12 ⩽ k ⩽ 12, -13 ⩽ l ⩽ 11	-13 ⩽ h ⩽ 13, -12 ⩽ k ⩽ 12, -13 ⩽ l ⩽ 12
Reflections collected	25510	12742	16024	9403
Independent reflections	6802 [R_{int} = 0.0350, R_{sigma} = 0.0358]	3454 [R_{int} = 0.0508, R_{sigma} = 0.0494]	4594 [R_{int} = 0.0434, R_{sigma} = 0.0448]	3341 [R_{int} = 0.1067, R_{sigma} = 0.0993]
Data/restraints/parameters	6802/2/371	3454/2/288	4594/2/270	3341/20/327
Goodness-of-fit on F^2	1.036	1.049	1.047	1.250
Final R indexes [I>=2σ (I)]	$R1^a$ = 0.0422, $wR2^b$ = 0.0888	$R1^a$ = 0.0559, $wR2^b$ = 0.1440	$R1^a$ = 0.0843, $wR2^b$ = 0.2280	$R1^a$ = 0.1023, $wR2^b$ = 0.2662

Final R indexes [all data]	R1[a] = 0.0516, wR2[b] = 0.0939	R1[a] = 0.0575, wR[b] = 0.1461	R1[a] = 0.0865, wR2[b] = 0.2316	R1 = 0.1236, wR2[b] = 0.2831
Largest diff. peak/hole / e Å^{-3}	0.71/-0.47	0.92/-0.58	1.10/-0.65	1.34/-0.91
Flack parameter	0.470(18)	0.41(6)	0.22(8)	0.28(11)

[a]$R_1=\Sigma||F_o|-|F_c||/\Sigma|F_o|$. [b]$wR_2= |\Sigma w(|F_o|^2-|F_c|^2)|/\Sigma|w(Fo^2)^2|^{1/2}$

Compounds	sql-(azpy)(pdia)-Ni-α_{DCM}	sql-(azpy)(pdia)-Ni-α_{PX}	sql-(azpy)(pdia)-Ni-α_{EB}
	(Open)	***(Open)***	***(Open)***
Identification code	2178495	2178498	2178496
Empirical formula	$C_{49}H_{38}N_{12}O_{10}Ni_2Cl_2$	$C_{28}H_{23}N_6O_5Ni$	$C_{28.68}N_6NiO_5H_{23.84}$
Formula weight	1143.23	582.23	591.19
Temperature/K	100(2)	100(2)	150(2)
Crystal system	monoclinic	monoclinic	monoclinic
Space group	*Pc*	*Pc*	*Pc*
a/Å	13.1981(4)	13.2041(5)	12.4808(15)
b/Å	10.1437(3)	10.1702(3)	10.1415(12)
c/Å	10.9639(3)	11.2100(4)	11.6631(14)
α /°	90	90	90
β /°	113.0830(10)	112.677(2)	95.591(3)
γ /°	90	90	90
Volume/Å^3	1350.30(7)	1389.00(9)	1469.2(3)
Z	1	2	2
ρ calcg/cm^3	1.406	1.392	1.336
μ /mm^{-1}	2.336	1.418	0.707
F(000)	586.0	602.0	611.3
Crystal size/mm^3	0.2 × 0.2 × 0.2	0.2 × 0.2 × 0.2	0.2 × 0.2 × 0.2
Radiation	CuKα (λ = 1.54178)	CuKα (λ = 1.54178)	MoKα (λ = 0.71073)
2Θ range for data collection/°	7.28 to 144.168	11.332 to 144.572	5.186 to 55.118
Index ranges	$-16 \leqslant h \leqslant 14, -12 \leqslant k \leqslant 12, -10 \leqslant l \leqslant 13$	$-16 \leqslant h \leqslant 16, -12 \leqslant k \leqslant 12, -13 \leqslant l \leqslant 13$	$-16 \leqslant h \leqslant 16, -13 \leqslant k \leqslant 12, -15 \leqslant l \leqslant 15$
Reflections collected	17356	14117	16248
Independent reflections	4143 [R_{int} = 0.0813, R_{sigma} = 0.0782]	4341 [R_{int} = 0.0468, R_{sigma} = 0.0509]	6209 [R_{int} = 0.0391, R_{sigma} = 0.0536]
Data/restraints/parameters	4143/2/323	4341/4/388	6209/69/347
Goodness-of-fit on F^2	1.078	1.040	1.041
Final R indexes [I>=2σ (I)]	R1^a = 0.0677, wR2^b = 0.1856	R1^a = 0.0651, wR2^b = 0.1730	R1^a = 0.0844, wR2^b = 0.2212
Final R indexes [all data]	R1^a = 0.0805, wR2^b = 0.1886	R1^a = 0.0692, wR2^b = 0.1788	R1^a = 0.1011, wR2^b = 0.2397
Largest diff. peak/hole / e Å^{-3}	2.01/-0.69	0.64/-0.94	2.45/-1.34
Flack parameter	0.45(5)	0.27(6)	0.38(4)

$^aR_1=\Sigma||F_o|-|F_c||/\Sigma|F_o|$. $^bwR_2=|\Sigma w(|F_o|^2-|F_c|^2)|/\Sigma|w(Fo^2)^2|^{1/2}$

Table S3. Comparative analysis of the sql layers differences between the six isolated phases (**α_{DMF}**, **β** and **α_{H2O}**, **α_{DCM}**, **α_{PX}**, **α_{EB}**) of **sql-(azpy)(pdia)-Ni**.

**sql-(azpy)(pdia)-Ni**	**α_{DMF}**	**β**	**α_{H2O}**
Distances between sql planes (gray) (Å)	5.120(6)	4.442(10)	4.827(14)
Dihedral angle of sql plane (gray) and inter layer Ni^{2+} cations plane (red) (°)	66.075(13) 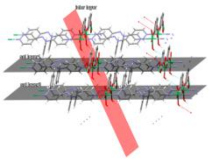	54.133(21) 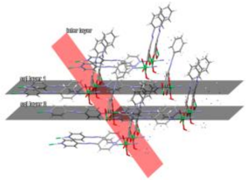	59.938(22) 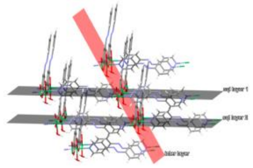
Length of hydrogen bond within layer (dO···O) (Å)	2.614(45)	2.618(59)	2.631(88)
Length of interlayer hydrogen bond (dO···O) (Å) 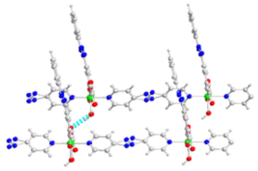	2.783(62)	2.832(68)	2.785(86)
Length of weak bond between guests and framework (Å). (blue dashed lines: hydrogen bond; green dashed lines: C-H···π bond)	d(O···H) = 2.516(3) 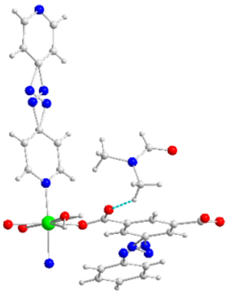	None 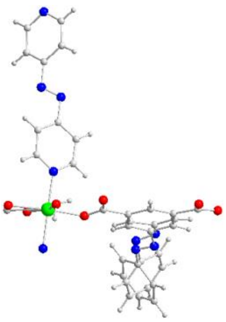	d(O···O) = 2.969(17) 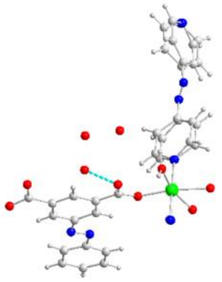

**sql-(azpy)(pdia)-Ni**	**α_{DCM}**	**α_{PX}**	**α_{EB}**
Distances between sql planes (gray) (Å)	5.043 (10)	5.172(31)	5.492 (15)
Dihedral angle of sql plane (gray) and interlayer Ni^{2+} cations plane (red) (°)	66.917(20) 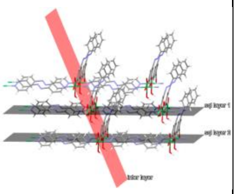	67.323(61) 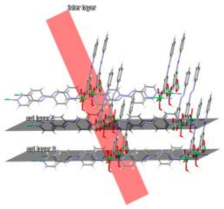	69.497(20) 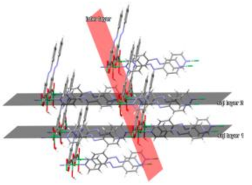
Length of hydrogen bond within layer (dO···O) (Å)	2.635(68)	2.596(70)	2.611(10)

Length of interlayer hydrogen bond (dO···O) (Å) 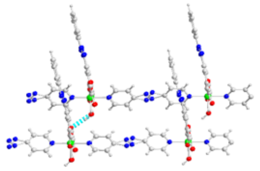	2.744(81)	2.791(91)	2.787(11)
Length of weak bond between guests and framework (Å). (blue dashed lines: hydrogen bond; green dashed lines: C-H···π bond)	d(H ··· O) = 2.294(50) 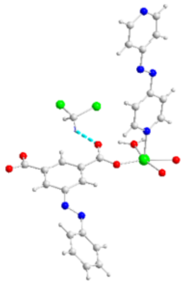	d(O···H) = 2.927(76)<br>d(C-H···π) = 3.065(1) 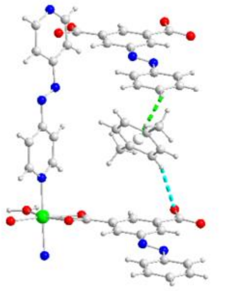	d(N···H) = 2.997(19)<br>d(C-H···π) = 3.005(2)<br>d(O···H) = 2.796(88) 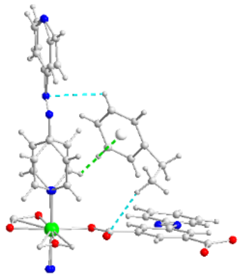

Table S4. Comparative analysis of the structural differences between the three isolated phases (**α_{DMF}**, **β** and **α_{H2O}**) of **sql-(azpy)(pdia)-Ni.**

		**sql-(azpy)(pdia)-Ni-α_{DMF}**	**sql-(azpy)(pdia)-Ni-β**	**sql-(azpy)(pdia)-Ni-α_{H2O}**
Coordination geometry	Bond length and bond angle	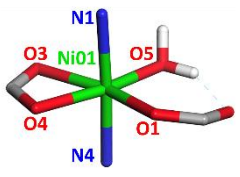	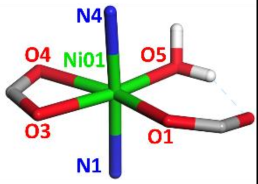	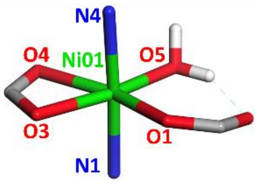
	Ni01-O1 (Å)	2.025(31)	2.033(33)	2.035(55)
	Ni01-O4 (Å)	2.105(31)	2.142(42)	2.146(52)
	Ni01-O3 (Å)	2.143(31)	2.126(38)	2.109(61)
	Ni01-O5 (Å)	2.043(31)	2.054(41)	2.046(55)
	Ni01-N1 (Å)	2.101(44)	2.128(59)	2.165(14)
	Ni01-N4 (Å)	2.081(72)	2.098(60)	2.106(12)
	∠O1-Ni01-N1 (°)	90.38(15)	89.17(17)	91.12(49)
	∠O4-Ni01-N1 (°)	89.16(15)	88.17(19)	92.06(49)
	∠O3-Ni01-N1 (°)	87.07(14)	88.22(19)	92.69(49)
	∠O5-Ni01-N1 (°)	92.58(16)	91.95(19)	86.16(47)
	∠O1-Ni01-O4 (°)	98.37(12)	105.93(13)[#1]	101.61(22)[#1]
	∠O4-Ni01-O3 (°)	62.57(12)	62.16(14)	62.29(21)
	∠O3-Ni01-O5 (°)	102.53(12)	98.03(16)[#2]	100.56(22)[#2]
	∠O5-Ni01-O1 (°)	96.62(12)	93.92(16)	95.58(24)
Dihedral angles between isophthalate ring plane (purple) and phenyl plane (blue) of ligand 1 (°)		8.161(24)<br>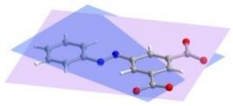	47.373(62),*<br>48.930(37)*<br>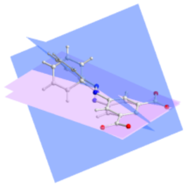	3.680(36)<br>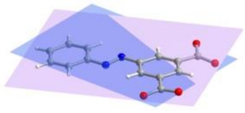
Dihedral angles between pyridyl plane 1 and pyridyl plane 2 of ligand 2 (°)		4.756(28)<br>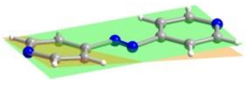	69.133(19)<br>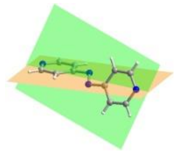	85.561(42),*<br>84.971(39)*<br>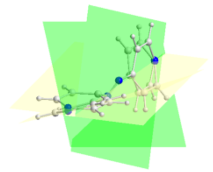
Dihedral angle of phenyl plane of ligand 1 (yellow and pink) and Ni atoms plane (green) (°)		77.639(25)<br>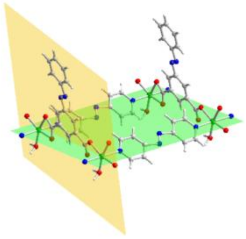	69.690(22),*<br>77.422(64),*<br>69.667 (31),*<br>77.286(80)*	83.387(30)<br>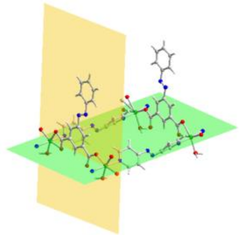

		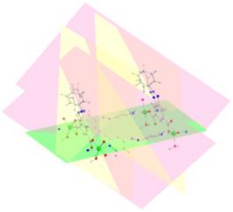	
sql net formed by ligand 1 and 2	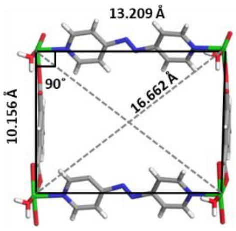	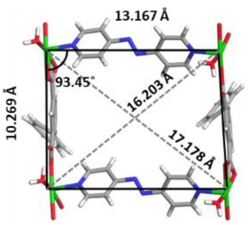	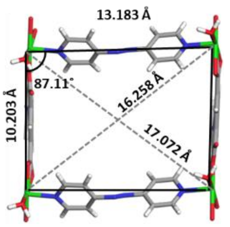
View along *c* axis of **sql-(azpy)(pdia)-Ni** sql nets	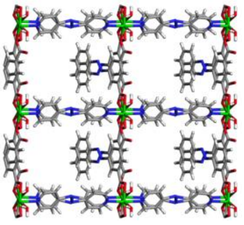	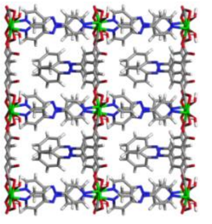	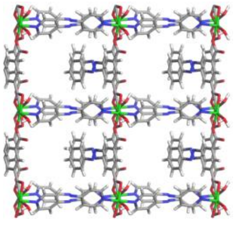

#1 The angle here is ∠O1-Ni01-O3 (°)

#2 The angle here is ∠O5-Ni01-O4 (°)

** The multiple values given come from the disorder of the two ligands.*

Table S5 Summary of flexible metal-organic materials exhibiting structural changes induced by water molecules.

Name	Dimension	Water sorption [a]	Ref.
**sql-(azpy)(pdia)-Ni**	2D	Y	This work
MIL-53as(ht)(Cr)	3D	N	9
MIL-53lt(Cr)	3D	N	10
$Co_2(bipy)_4(SO_4)_2(CH_3OH)]$	3D	N	11
$Ba_3(H_2O)_3(L)_2(H_2O)_8$	3D	N	12
UWDM-1	3D	N	13
UTSA-300-Cu / NCU-100	2D	N	14
MIL-53(Al)	3D	N	15
SHF-61	3D	N	16
$\{[Ln(TCMBT)(H_2O)_3]\cdot 4H_2O\}_n$ / $\{[Ln(TCMBT)(C_2H_5OH)\text{-}(H_2O)_2]\cdot 2.5H_2O\}_n$	3D	N	17
MIL-88	3D	N	18
Cu(BDTri)(DMF)	3D	N	19
MUV-2	3D	N	20
NTU-19, NTU-20, NTU-21, NTU-22	2D	N	21
AEMOF-1	3D	N	22
$[Cu(HL)(DMSO)\cdot(MeOH)]_n$ / $H_3L\cdot 0.5[Cu_2\text{-}(OH)_4\cdot 6H_2O]\cdot 4H_2O(OH)_4\cdot 6H_2O]\cdot 4H_2O$	3D	N	23
$(NH_4)_3[Co_2(bamdpH)_2(HCOO)(H_2O)_2]$ / $[Co(bamdpH_2)(H_2O)_2]\cdot 2H_2O$ / $[Co\text{-}(bamdpH_2)(H_2O)_2]\cdot H_2O$	1D	N	24
$[Cu(tzc)(dpp)]n\cdot 2H_2O$ / $[Cu(tzc)(dpp)]_n$ (6I, 6II, 6III)	1D	N	25
$[Cu_2(pzdc)_2(dpyg)]_n$	3D	N	26
JUK-8	3D	Y	27
DUT-98	3D	Y	28
SIFSIX-23-Cu	3D	Y	29
CID-5,CID-6,CID-5/6	2D	Y	30
CPL-2	3D	Y	31
MIL-53(Cr)	3D	Y	32
$\{[Ce(tci)]\}_n$	3D	Y	33
Co MOF-BP and RP	3D	Y	34
$\{[Zn(4\text{-}bpdh)(DHT)]\cdot(MeOH)(H_2O)\}_n$	3D	Y	35
MIL-88A@PVDF	3D	Y	36
$[Zn(H_2SSA)_2\text{-}(H_2O)_2]\cdot 7.1H_2O$ / $[Zn(H_2O)_6]\cdot 2H_2SSA$ $4H_2O$	0D	Y	37
$(Me_2NH_2)[Eu(L)]$	2D	Y	38
BUT-8(Cr)A	3D	Y	39
ZPF-2-Co	3D	Y	40

MIL-53(M) (M=Al, Fe)	3D	Y	[41]

[a] *Y - water sorption investigated, N - water sorption not investigated.*

Totally 34 studies reported structural changes triggered by water molecules, 15 of these did water sorption investigations.

References

(1) Blatov, V. A.; Shevchenko, A. P.; Proserpio, D. M. Applied Topological Analysis of Crystal Structures with the Program Package Topospro. *Cryst. Growth Des.* **2014**, *14* (7), 3576–3586.

(2) Groom, C. R.; Bruno, I. J.; Lightfoot, M. P.; Ward, S. C. The Cambridge Structural Database. *Acta Crystallogr. Sect. B Struct. Sci. Cryst. Eng. Mater.* **2016**, *72* (2), 171–179.

(3) Gong, C. Bin; He, L. H.; Long, J. F.; Liu, L. T.; Liu, S.; Tang, Q.; Fu, X. K. Synthesis and Characterisation of Azobenzene-Bridged Cationic-Cationic and Neutral-Cationic Electrochromic Materials. *Synth. Met.* **2016**, *220*, 147–154.

(4) He, H.; Du, J.; Su, H.; Yuan, Y.; Song, Y.; Sun, F. Four New Metal-Organic Frameworks Based on Bi-, Tetra-, Penta-, and Hexa-Nuclear Clusters Derived from 5-(Phenyldiazenyl)Isophthalic Acid: Syntheses, Structures and Properties. *CrystEngComm* **2015**, *17* (5), 1201–1209.

(5) Sheldrick, G. M.; Bruker, A. X. S. Inc., Madison, WI, 2000;(b) GM Sheldrick. *Acta Crystallogr., Sect. A Fundam. Crystallogr* **2015**, *71*, 3–8.

(6) Krause, L.; Herbst-Irmer, R.; Sheldrick, G. M.; Stalke, D. Comparison of Silver and Molybdenum Microfocus X-Ray Sources for Single-Crystal Structure Determination. *J. Appl. Crystallogr.* **2015**, *48* (1), 3–10.

(7) Sheldrick, G. M. Crystal Structure Refinement with SHELXL. *Acta Crystallogr. Sect. C Struct. Chem.* **2015**, *71* (Md), 3–8.

(8) Dolomanov, O. V.; Bourhis, L. J.; Gildea, R. J.; Howard, J. A. K.; Puschmann, H. OLEX2: A Complete Structure Solution, Refinement and Analysis Program. *J. Appl. Crystallogr.* **2009**, *42* (2), 339–341.

(9) Millange, F.; Serre, C.; Férey, G. Synthesis, Structure Determination and Properties of MIL-53as and MIL-53ht: The First Criii Hybrid Inorganic–Organic Microporous Solids: Criii(OH)·{O2C–C6H4–CO2}·{HO2C–C6H4–CO2H}x. *Chem. Commun.* **2002**, No. 8, 822–823.

(10) Serre, C.; Millange, F.; Thouvenot, C.; Noguès, M.; Marsolier, G.; Louër, D.; Férey, G. Very Large Breathing Effect in the First Nanoporous Chromium(III)-Based Solids: MIL-53 or CrIII(OH)·{O2C-C6H4- CO2}·{HO2C-C6H4 -CO2H}x·H2Oy. *J. Am. Chem. Soc.* **2002**, *124* (45), 13519–13526.

(11) Bradshaw, D.; Warren, J. E.; Rosseinsky, M. J. Reversible Concerted Ligand Substitution at Alternating Metal Sites in an Extended Solid. *Science.* **2007**, *315* (5814), 977–980.

(12) Chandler, B. D.; Enright, G. D.; Udachin, K. A.; Pawsey, S.; Ripmeester, J. A.; Cramb, D. T.; Shimizu, G. K. H. Mechanical Gas Capture and Release in a Network Solid via Multiple Single-Crystalline Transformations. *Nat. Mater.* **2008**, *7* (3), 229–235.

(13) Vukotic, V. N.; Harris, K. J.; Zhu, K.; Schurko, R. W.; Loeb, S. J. Metal-Organic Frameworks with Dynamic Interlocked Components. *Nat. Chem.* **2012**, *4* (6), 456–460.

(14) Wang, J.; Zhang, Y.; Zhang, P.; Hu, J.; Lin, R. B.; Deng, Q.; Zeng, Z.; Xing, H.; Deng, S.; Chen, B. Optimizing Pore Space for Flexible-Robust Metal-Organic Framework to Boost Trace Acetylene Removal. *J. Am. Chem. Soc.* **2020**, *142* (21), 9744–9751.

(15) Loiseau, T.; Serre, C.; Huguenard, C.; Fink, G.; Taulelle, F.; Henry, M.; Bataille, T.; Férey, G. A Rationale for the Large Breathing of the Porous Aluminum Terephthalate (MIL-53) Upon Hydration. *Chem. A Eur. J.* **2004**, *10* (6), 1373–1382.

(16) Carrington, E. J.; McAnally, C. A.; Fletcher, A. J.; Thompson, S. P.; Warren, M.; Brammer, L. Solvent-Switchable Continuous-Breathing Behaviour in a Diamondoid Metal-Organic Framework and Its Influence on CO2 versus CH4 Selectivity. *Nat. Chem.* **2017**, *9* (9), 882–889.

(17) Sun, R.; Wang, S.; Xing, H.; Bai, J.; Li, Y.; Pan, Y.; You, X. Unprecedented 4264 Topological 2-D Rare-Earth Coordination Polymers from a Flexible Tripodal Acid with Additional Amide Groups. *Inorg. Chem.* **2007**, *46* (21), 8451–8453.

(18) Horcajada, P.; Salles, F.; Wuttke, S.; Devic, T.; Heurtaux, D.; Maurin, G.; Vimont, A.; Daturi, M.; David, O.; Magnier, E.; Stock, N.; Filinchuk, Y.; Popov, D.; Riekel, C.; Férey, G.; Serre, C. How Linker's Modification Controls Swelling Properties of Highly Flexible Iron(III) Dicarboxylates MIL-88. *J. Am. Chem. Soc.* **2011**, *133* (44), 17839–17847.

(19) Demessence, A.; Long, J. R. Selective Gas Adsorption in the Flexible Metal-Organic Frameworks Cu(BDTri)L (L = DMF, DEF). *Chem. A Eur. J.* **2010**, *16* (20), 5902–5908.

(20) Souto, M.; Romero, J.; Calbo, J.; Vitórica-Yrezábal, I. J.; Zafra, J. L.; Casado, J.; Ortí, E.; Walsh, A.; Mínguez Espallargas, G. Breathing-Dependent Redox Activity in a Tetrathiafulvalene-Based Metal-Organic Framework. *J. Am. Chem. Soc.* **2018**, *140* (33), 10562–10569.

(21) Wang, H.; Cao, H.; Zheng, J. J.; Mathew, S.; Hosono, N.; Zhou, B.; Lyu, H.; Kusaka, S.; Jin, W.; Kitagawa, S.; Duan, J. Finely Controlled Stepwise Engineering of Pore Environments and Mechanistic Elucidation of Water-Stable, Flexible 2D Porous Coordination Polymers. *Chem. - A Eur. J.* **2018**, *24* (24), 6412–6417.

(22) Douvali, A.; Tsipis, A. C.; Eliseeva, S. V.; Petoud, S.; Papaefstathiou, G. S.; Malliakas, C. D.; Papadas, I.; Armatas, G. S.; Margiolaki, I.; Kanatzidis, M. G.; Lazarides, T.; Manos, M. J. Turn-on Luminescence Sensing and Real-Time Detection of Traces of Water in Organic Solvents by a Flexible Metal-Organic Framework. *Angew. Chem. Int. Ed.* **2015**, *54* (5), 1651–1656.

(23) Nakatsuka, S.; Watanabe, Y.; Kamakura, Y.; Horike, S.; Tanaka, D.; Hatakeyama, T. Solvent-Vapor-Induced Reversible Single-Crystal-to-Single-Crystal Transformation of a Triphosphaazatriangulene-Based Metal–Organic Framework. *Angew. Chem. Int. Ed.* **2020**, *59* (4), 1435–1439.

(24) Cai, Z. S.; Bao, S. S.; Wang, X. Z.; Hu, Z.; Zheng, L. M. Multiple-Step Humidity-Induced Single-Crystal to Single-Crystal Transformations of a Cobalt Phosphonate: Structural and Proton Conductivity Studies. *Inorg. Chem.* **2016**, *55* (7), 3706–3712.

(25) Wriedt, M.; Yakovenko, A. A.; Halder, G. J.; Prosvirin, A. V.; Dunbar, K. R.; Zhou, H. C. Reversible Switching from Antiferro- to Ferromagnetic Behavior by Solvent-Mediated, Thermally-Induced Phase Transitions in a Trimorphic Mof-Based Magnetic Sponge System. *J. Am. Chem. Soc.* **2013**, *135* (10), 4040–4050.

(26) Kitaura, R.; Fujimoto, K.; Noro, S.; Kondo, M.; Kitagawa, S. A Pillared-Layer Coordination Polymer Network Displaying Hysteretic Sorption: [Cu2(Pzdc)2(Dpyg)]n (Pzdc= Pyrazine-2,3-Dicarboxylate; Dpyg=1,2-Di(4-Pyridyl)Glycol). *Angew. Chem. Int. Ed.* **2002**, *41* (1), 133–135.

(27) Roztocki, K.; Formalik, F.; Krawczuk, A.; Senkovska, I.; Kuchta, B.; Kaskel, S.; Matoga, D. Collective Breathing in an Eightfold Interpenetrated Metal–Organic Framework: From Mechanistic Understanding towards Threshold Sensing Architectures. *Angew. Chem. Int. Ed.* **2020**, *59* (11), 4491–4497.

(28) Krause, S.; Bon, V.; Du, H.; Dunin-Borkowski, R. E.; Stoeck, U.; Senkovska, I.; Kaskel, S. The Impact of Crystal Size and Temperature on the Adsorption-Induced Flexibility of the Zr-Based Metal-Organic Framework DUT-98. *Beilstein J. Nanotechnol.* **2019**, *10*, 1737–1744.

(29) Song, B. Q.; Yang, Q. Y.; Wang, S. Q.; Vandichel, M.; Kumar, A.; Crowley, C.; Kumar, N.; Deng, C. H.; Gasconperez, V.; Lusi, M.; Wu, H.; Zhou, W.; Zaworotko, M. J. Reversible Switching between Nonporous and Porous Phases of a New SIFSIX Coordination Network Induced by a

Flexible Linker Ligand. *J. Am. Chem. Soc.* **2020**, *142* (15), 6896–6901.
(30) Fukushima, T.; Horike, S.; Inubushi, Y.; Nakagawa, K.; Kubota, Y.; Takata, M.; Kitagawa, S. Solid Solutions of Soft Porous Coordination Polymers: Fine-Tuning of Gas Adsorption Properties. *Angew. Chem. Int. Ed.* **2010**, *49* (28), 4820–4824.
(31) Matsuda, R.; Kitaura, R.; Kitagawa, S.; Kubota, Y.; Kobayashi, T. C.; Horike, S.; Takata, M. Guest Shape-Responsive Fitting of Porous Coordination Polymer with Shrinkable Framework. *J. Am. Chem. Soc.* **2004**, *126* (43), 14063–14070.
(32) Bourrelly, S.; Moulin, B.; Rivera, A.; Maurin, G.; Devautour-Vinot, S.; Serre, C.; Devic, T.; Horcajada, P.; Vimont, A.; Clet, G.; Daturi, M.; Lavalley, J. C.; Loera-Serna, S.; Denoyel, R.; Llewellyn, P. L.; Férey, G. Explanation of the Adsorption of Polar Vapors in the Highly Flexible Metal Organic Framework MIL-53(Cr). *J. Am. Chem. Soc.* **2010**, *132* (27), 9488–9498.
(33) Ghosh, S. K.; Zhang, J. P.; Kitagawa, S. Reversible Topochemical Transformation of a Soft Crystal of a Coordination Polymer. *Angew. Chem. Int. Ed.* **2007**, *46* (42), 7965–7968.
(34) Chen, Q.; Chang, Z.; Song, W. C.; Song, H.; Song, H. Bin; Hu, T. L.; Bu, X. H. A Controllable Gate Effect in Cobalt(II) Organic Frameworks by Reversible Structure Transformations. *Angew. Chem. Int. Ed.* **2013**, *52* (44), 11550–11553.
(35) Bhattacharya, B.; Halder, A.; Paul, L.; Chakrabarti, S.; Ghoshal, D. Eye-Catching Dual-Fluorescent Dynamic Metal–Organic Framework Senses Traces of Water: Experimental Findings and Theoretical Correlation. *Chem. A Eur. J.* **2016**, *22* (42), 14998–15005.
(36) Troyano, J.; Carné-Sánchez, A.; Pérez-Carvajal, J.; León-Reina, L.; Imaz, I.; Cabeza, A.; Maspoch, D. A Self-Folding Polymer Film Based on Swelling Metal–Organic Frameworks. *Angew. Chem. Int. Ed.* **2018**, *57* (47), 15420–15424.
(37) Song, J. H.; Kim, D. W.; Kang, D. W.; Lee, W. R.; Hong, C. S. Humidity-Triggered Single-Crystal-to-Single-Crystal Structural Transformations in a Zn(Ii) Coordination Polymer Displaying Unusual Activation Energy Change in Proton Conductivity. *Chem. Commun.* **2019**, *55* (65), 9713–9716.
(38) Wei, Y. S.; Hu, X. P.; Han, Z.; Dong, X. Y.; Zang, S. Q.; Mak, T. C. W. Unique Proton Dynamics in an Efficient MOF-Based Proton Conductor. *J. Am. Chem. Soc.* **2017**, *139* (9), 3505–3512.
(39) Yang, F.; Xu, G.; Dou, Y.; Wang, B.; Zhang, H.; Wu, H.; Zhou, W.; Li, J. R.; Chen, B. A Flexible Metal-Organic Framework with a High Density of Sulfonic Acid Sites for Proton Conduction. *Nat. Energy* **2017**, *2* (11), 877–883.
(40) Yang, M.; Wang, S. Q.; Liu, Z.; Chen, Y.; Zaworotko, M. J.; Cheng, P.; Ma, J. G.; Zhang, Z. Fabrication of Moisture-Responsive Crystalline Smart Materials for Water Harvesting and Electricity Transduction. *J. Am. Chem. Soc.* **2021**, *143* (20), 7732–7739.
(41) Shigematsu, A.; Yamada, T.; Kitagawa, H. Wide Control of Proton Conductivity in Porous Coordination Polymers. *J. Am. Chem. Soc.* **2011**, *133* (7), 2034–2036.
